# Nutrients and Other Environmental Factors Influence Virus Abundances across Oxic and Hypoxic Marine Environments

**DOI:** 10.3390/v9060152

**Published:** 2017-06-17

**Authors:** Jan F. Finke, Brian P. V. Hunt, Christian Winter, Eddy C. Carmack, Curtis A. Suttle

**Affiliations:** 1 Department of Earth, Ocean and Atmospheric Sciences, University of British Columbia, Vancouver, BC V6T 1Z4, Canada; jfinke@eos.ubc.ca (J.F.F.); b.hunt@oceans.ubc.ca (B.P.V.H.); christian.winter@univie.ac.at (C.W.); 2 Institute for the Oceans and Fisheries, University of British Columbia, Vancouver, BC V6T 1Z4, Canada; 3 Hakai Institute, P.O. Box 309, Heriot Bay, BC, Canada; 4 Fisheries and Oceans Canada, Institute of Ocean Sciences, Sidney, BC V8L 4B2, Canada; Eddy.Carmack@dfo-mpo.gc.ca; 5 Department of Botany, University of British Columbia, Vancouver, BC V6T 1Z4, Canada; 6 Department of Microbiology and Immunology, University of British Columbia, Vancouver, BC V6T 1Z3, Canada

**Keywords:** viral abundance, environmental variables, multivariate model, Akaike Information Criterion

## Abstract

Virus particles are highly abundant in seawater and, on average, outnumber microbial cells approximately 10-fold at the surface and 16-fold in deeper waters; yet, this relationship varies across environments. Here, we examine the influence of a suite of environmental variables, including nutrient concentrations, salinity and temperature, on the relationship between the abundances of viruses and prokaryotes over a broad range of spatial and temporal scales, including along a track from the Northwest Atlantic to the Northeast Pacific via the Arctic Ocean, and in the coastal waters of British Columbia, Canada. Models of varying complexity were tested and compared for best fit with the Akaike Information Criterion, and revealed that nitrogen and phosphorus concentrations, as well as prokaryote abundances, either individually or combined, had significant effects on viral abundances in all but hypoxic environments, which were only explained by a combination of physical and chemical factors. Nonetheless, multivariate models of environmental variables showed high explanatory power, matching or surpassing that of prokaryote abundance alone. Incorporating both environmental variables and prokaryote abundances into multivariate models significantly improved the explanatory power of the models, except in hypoxic environments. These findings demonstrate that environmental factors could be as important as, or even more important than, prokaryote abundance in describing viral abundance across wide-ranging marine environments.

## 1. Introduction

Viruses play an important role in aquatic ecosystems, which includes influencing host diversity and the flux of nutrients and carbon through the viral shunt [1]. They are highly abundant, typically ranging in concentration across different environments from 10^6^ mL^−1^ to as high as 10^8^ mL^−1^ [2,3,4], with generally lower abundances in the deep sea and higher abundances at productive coastal sites. Because contact rates between viruses and their potential hosts are proportional to viral abundance, higher densities of viruses generally lead to a greater impact on microbial host populations [5,6]. Given that the most abundant host cells for viruses in the oceans are prokaryotes, and that these are largely bacteria, prokaryotes will henceforth be referred to as bacteria. 

Over the years, it has been established that viral abundance is about an order of magnitude higher than bacterial abundance [7], but the virus to bacteria ratio (VBR) varies greatly among host-virus systems and environments [8,9,10,11]. In a meta-analysis of 25 studies, Wigington et al. [10] found that the VBR ranged from 10.5 to 16. They also demonstrated the limitation of models using a fixed VBR ratio of 10:1, and applied non-linear power functions to relate viral and bacterial abundances. Conversely, Knowles et al. [9] showed a linear correlation between viral and bacterial abundances across a range of habitats, and that there was a relative decrease in the relationship with increasing bacterial abundance. From Wigington et al. [10] and Knowles et al. [9], it is apparent that the relationship between viral and bacterial abundances varies substantially among studies. Additionally, there were significant differences in correlations between bacterial and viral abundances in samples from lakes, the upper Pacific, deep Pacific and Arctic oceans [11]. Observations that the VBR varies under different conditions and among locations implies that it could be affected by environmental variables, with burst size, viral decay rates and photosynthetic host density potentially affecting the VBR [10,11,12]. Hence, while viral and bacterial abundances for specific studies or locations are typically highly correlated, deriving relationships that extend across biomes requires models that include environmental variables that affect the virus–host relationship.

Temperature and salinity are environmental variables that can directly affect virus–host interactions. For example, in the microalgae *Phaeocystis globosa* and *Heterosigma akashiwo*, lysis of infected cells occurred over a narrow temperature range and the different viruses were inactivated above temperatures ranging from 20 to 35 °C [13,14]. A similar pattern of inactivation at 40 °C was shown for a phage of the marine prokaryote *Pseudoalteromonas marina* [15]. Inactivation temperatures for marine viruses, however, are usually above 20 °C, which is higher than that which many virus–host systems are likely to encounter in temperate and Arctic waters, but can play a role in microenvironments in temperate waters. Furthermore, a rise in temperatures can favor the switch from a lysogenic to a lytic cycle in a marine phage–host system [16], which would affect the total community viral production. Salinity has also been shown to interfere with the initial step of viral infection; salt concentrations above 3 M NaCl lowered infectivity and adsorption in a marine bacteria–virus system in culture [17]. Additionally, marine phages can require salt for particle stability [18]. However, another study showed an increase in viral abundance and drastic change in the viral community composition at hypersaline conditions above 240 practical salinity units (PSU) [19]. 

Light can also influence virus-host interactions in both positive and negative ways. Photosynthetically active radiation (PAR) is required for phytoplankton growth, and is thus crucial for replication of phytoplankton viruses. Even adsorption of viral particles to their host can be light dependent [20], as can be the duration of the viral replication cycle and the burst size [21,22]. Yet, some viruses infecting phytoplankton, including those infecting *H. akashiwo*, appear to be less sensitive to changes in the light regime [23,24]. Nonetheless, the final stage of virus replication is very energy demanding, and can be especially vulnerable to light limitation in photosynthetic hosts [25]. Light can also have highly negative effects on viral replication. For example, UV radiation is a major factor causing viral decay, and decay rates for viruses of bacteria, cyanobacteria and eukaryotic phytoplankton increase in proportion to irradiance [5,26,27,28]. In the ocean, light effects are restricted to the upper photic zone, with PAR influencing interactions of viruses of photosynthetic hosts, and UV radiation causing decay of all viruses. 

Nutrients also have profound effects on virus–host interactions. Since viral particles mainly consist of a genome and a capsid, they have a different stoichiometric composition than cellular organisms. A recent study [29] calculated that the C:N:P stoichiometry of viruses is about 17:6:1, which is very different from that of their cellular hosts, which is typically 69:16:1 for heterotrophs and 106:16:1 for phototrophs [29,30,31]. Moreover, up to 87% of cellular phosphorus can be assimilated into viral particles during replication, highlighting the relatively high demand of viruses for nitrogen and phosphorus, and the importance of these nutrients for viral replication [29]. For example, phosphorus depletion can result in reduced viral production for a variety of prymnesiophytes and their viruses [32,33], and production of viruses infecting *Emiliania huxleyi* were affected by phosphate and nitrate availability [34]. In turn, phosphate addition can increase viral production [35]. The limited available data indicate that nitrogen limitation either has no impact, or reduces viral production [32,36]. Moreover, there is mounting evidence that hosts and viruses adapt to environmental conditions [37]. In summary, environmental factors affect viral replication, and thus would be expected to affect the relationship between virus and bacterial abundances. 

Despite the highlighted importance of environmental factors to virus–host interactions, their relationship to the relative abundances of viruses and bacteria in the environment has not been rigorously explored. This study addresses these influences by exploring which environmental variables influence the relative abundances of viruses and bacteria across a wide range of samples derived from diverse environments. This approach allows better predictions of how environmental differences affect the relative abundances of viruses and bacteria.

## 2. Materials and Methods

**Figure 1:**
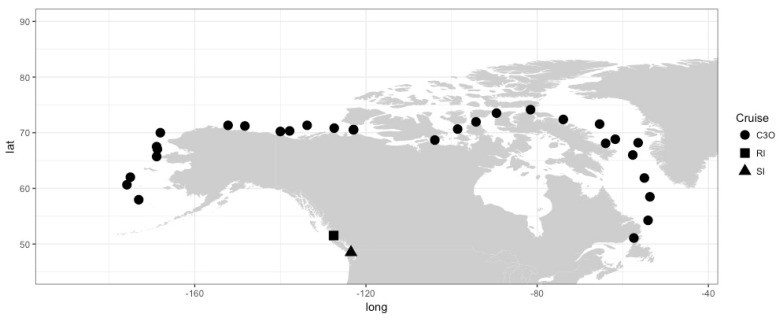
Map of sampling locations by project. Each location represents multiple depths and/or time points. C3O: Canada’s Three Oceans project; lat: Latitude; long: Longitude; RI: Rivers Inlet; SI: Saanich Inlet.

Data from 515 samples were compiled from several years of data collected in Saanich Inlet (SI; 48°35′ N, 123°30′ W) and Rivers Inlet (RI; 51°26′ N, 127°38′ W) [38], BC, Canada, as well as along a cruise track from the Labrador Sea to the coast of British Columbia through the Arctic Ocean as part of the Canada’s Three Oceans project (C3O) [39] (Figure 1). Water samples from depth profiles were collected with Go-Flo bottles and subsampled for various analyses, as detailed below. Samples were taken from surface waters to a maximum depth of 1000 m.

Abundances of double-stranded DNA (dsDNA) viruses and bacteria were determined in duplicate water samples using a Beckton Dickinson FACSCalibur flow cytometer (Franklin Lakes, NJ, USA) with a 15 mW 488 nm air-cooled argon ion laser, as described in [40]. Briefly, samples were fixed for 15 min at 4 °C in the dark with electron microscopy-grade glutaraldehyde (25%; Sigma-Aldrich, Saint Louis, MO, USA), final concentration 0.5%, followed by snap-freezing in liquid nitrogen and storage at −80 °C. Right before analysis, the samples are thawed and diluted in 0.2 μm filtered, autoclaved 10:1 TE buffer (10 mM Tris HCl; 1 mM ethylenediaminetetraacetic acid (EDTA) pH 8.0) and stained with SYBR Green I (Invitrogen, Carlsbad, CA, USA) at a final concentration of 0.5 × 10^−4^ of the commercial stock, for 10 min at 80 °C in a water bath. Samples were diluted in TE buffer (pH 8.0), if necessary, to reach 100 to 1000 events s^−1^. Viruses were discriminated by plotting green fluorescence against side scatter, and the results analyzed with CYTOWIN version 4.31 [41].

Nutrient samples were filtered through 0.22 µm pore-size polyvinylidene difluoride (PVDF) syringe filters and stored at −20 °C till analysis. Total nitrate (NO_3_) (reduced to nitrite) and nitrite (referred to as the predominant nitrate hereafter), phosphate (PO_4_) and silicate (SiO_4_) were analyzed with a Bran & Luebbe AutoAnalyzer 3 (Norderstedt, Germany) using air-segmented continuous-flow analysis. Colorimetry was used to measure the concentrations of reduced nitrate [42] and silicate at 550 nm, and reduced orthophosphate [43] at 880 nm. 

For physical data, in situ profiles of temperature, salinity and depth were measured with a SBE 25 (SI and RI) or SBE 911 (C3O) CTD (Seabird Electronics, Inc., Bellevue, WA, USA). Chlorophyll concentration was estimated by a fast-repetition-rate fluorometer (FRRF), for SI and RI a WetStar fluorometer (Seabird Electronics, Inc., Bellevue, WA, USA) for C3O a Seapoint Chlorophyll Fluorometer (Seapoint Sensors, Exeter, NH, USA), mounted to the CTD. Fluorescence data were converted to chlorophyll concentrations based on standard curves. These curves were derived from measurements of in situ fluorescence, as well as extracted chlorophyll concentrations made on samples from a range of environments. Oxygen was measured with a SBE 43 oxygen sensor and PAR was measured with a QSP-200PD (SI and RI) or QSP-2300 (C3O) profiling sensor (Biospherical Instruments, San Diego, CA, USA).

Of the 515 samples, 47 samples from Saanich Inlet were missing bacterial counts, and 211 samples from Rivers Inlet did not have PAR data; these were left out of the analysis when applicable. Other irregularly missing data points, with <10% missing per variable, were filled with weighted data by multiple imputation, a statistical technique to analyze data sets with missing values. The data were divided into the following three subsets: “Arctic”, including sub-Arctic samples from the Atlantic and Pacific; “inlet”; and “hypoxic”. Data from Saanich Inlet and Rivers Inlet comprised the inlet subset; data from C3O made up the Arctic subset; and all samples with an oxygen concentration below 1.5 mL·L^−1^ [44] were pooled into the hypoxic subset. Statistical analysis was done in the programming language, R [45]. A linear discriminant analysis (LDA) of the samples based on scaled environmental variables was performed with the MASS package (version 7.3-40) to confirm the prior classification of samples into environments. Input variables for the LDA were temperature, salinity, chlorophyll, nitrate, phosphate, silicate and oxygen. Samples for one sampling day and one site were removed from the inlet subset due to extremely high viral counts, exceeding 1.5 times the interquartile range, and were thus considered to be outliers. Temperature, salinity and chlorophyll were log transformed to compensate for outliers and approximate normal distribution. Viral and bacterial abundances were log_10_ transformed. Transformations were kept consistent across sub-sets of data so that the models were comparable. The data were explored for normal distributions in histogram plots and Pearson correlation coefficients were used to explore variables for patterns of collinearity (Figures S1–S3).

Single variable correlations were measured using linear models with log_10_ transformed viral and bacterial abundances, while nitrate and phosphate data were not transformed. The explanatory power of the models was expressed as the coefficient of determination (R^2^) and significances in *p*-values; the slope of the regression is also given. Multivariate regressions were determined with generalized linear models (GLM), with a Gaussian distribution and an identity link function being run for log_10_ transformed viral abundance against environmental variables and/or log_10_ transformed bacterial abundance using the MASS package [46]. Models were run at a range of complexities, ranging from one input variable to all possible variables. For each complexity, the optimal combination of variables was selected based on the Akaike Information Criterion (AIC) with the Stats package [45]. Optimal models were then selected by comparing the AICs and considering improvements in explanatory power at different complexities; a relative drop in the AIC of two was considered relevant. Model fit was tested with a combined McFadden pseudo R^2^, and significance was tested on *z*-values per coefficient. Pseudo R^2^s were determined with the BaylorEdPsych (version 0.5) package [47]. The use of GLMs and model selection based on the AIC was done to account for deviations from a normal distribution in the variable and to reduce model complexity to significant predictors. Multicollinearity of predictors in the models was assessed by the Variance Inflation Factor (VIF), collinear predictors were then removed from the models, retaining only one. Models were assessed for their homogeneity of variance and the normal distribution of residuals, additionally the normal distribution of residuals was tested with the Shapiro–Wilk test.

## 3. Results

The data used in this study are categorized into “inlet” samples from Saanich and Rivers Inlets, “hypoxic” samples, mainly from deep inlet water, and “Arctic” samples from the Canadian Arctic and sub-Arctic; each environmental category has distinguishing environmental conditions. 

Viral abundance data that went into models ranged from 4.83 × 10^5^ to 1.40 × 10^8^ viruses mL^−1^, and bacterial abundances ranged from 7.31 × 10^4^ to 7.40 × 10^7^ bacteria mL^−1^ (Table 1). A set of outlier samples from June 2009 in Rivers Inlet had extraordinarily high viral abundances with 1.40 × 10^8^ viruses mL^−1^ at 10 m, which remained above 4 × 10^7^ viruses mL^−1^ until 320 m depth. Bacterial abundances were proportionally high and varied between 7.4 × 10^7^ and 2.04 × 10^7^ bacteria mL^−1^ over the same depths, but the environmental variables did not show a correlated pattern.

The range in environmental data was also large. Temperature ranged from −2 to 15 °C and salinity from 3 to 35 PSU, while chlorophyll and oxygen ranged from 0.03 to 44 mg·m^−3^ and from 0.005 to 10 mL·L^−1^, respectively. PAR data, which was only available for Saanich Inlet and C3O had a maximum of 669 µmol quanta m^−2^·s^−1^ at the surface and was undetectable in hypoxic waters in Saanich Inlet. Nutrient values ranged from 0.01 to 54 µM for nitrate, 0.006 to 7 µM for phosphate and 0.07 to 141 µM for silicate. After classifying the data into the three environments and appropriate transformations, the data generally demonstrated normal distribution. However, even after log transformation, temperature and salinity in some environments were somewhat skewed (Figures S1–S3). Correlating all environmental variables, especially nutrient data in the inlet environment, showed some degree of collinearity based on the Pearson correlation coefficient (Figures S1–S3). Variables displaying collinearity in the multivariate models based on the VIF were subsequently reduced to one variable.

### 3.1. Samples Can Be Classified into Environments

**Figure 2:**
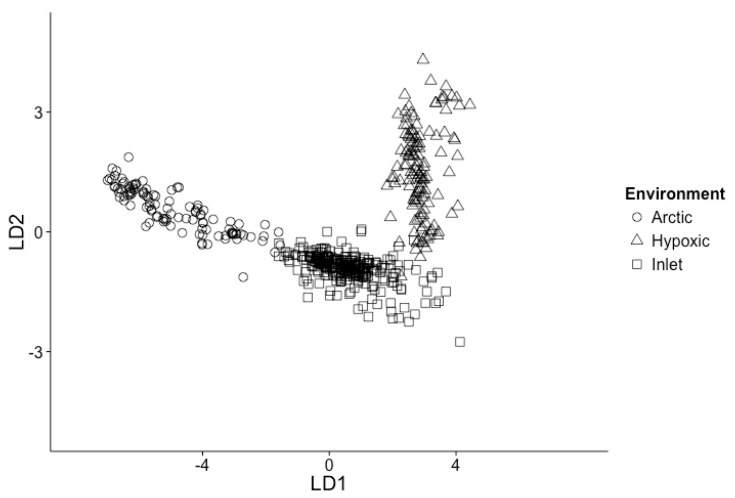
Linear discriminant analysis of samples used in models, based on temperature, salinity, nitrate, phosphate, silicate, chlorophyll and oxygen. Arctic samples (open circles); Inlet samples (open squares); Hypoxic samples (open triangles).

Linear discriminant analysis (LDA) of all samples based on scaled environmental data, consisting of temperature, salinity, oxygen, nitrate, phosphate, silicate and chlorophyll, supported the classification of the data into three groups (Figure 2), reflecting Arctic, inlet and hypoxic environments. The first dimension LD1 describes 92.6% of the variation and the second dimension LD2 7.4%, with temperature and phosphate concentrations being the strongest components. The environments form well-defined clusters, with the Arctic and inlet samples partially overlapping and the hypoxic samples a clearly separated. 

**Figure 3:**
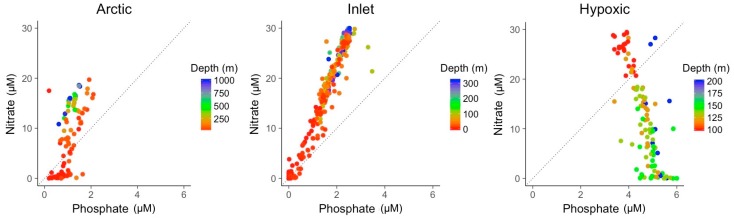
Nitrate to phosphate ratio for the samples from the three different environments. Colors indicate the sampling depth. The dashed line indicates the elemental 5:1 stoichiometric N:P ratio of viral particles.

Besides their variability in temperature and salinity, the three environments varied markedly in the concentrations of nitrate and phosphate (Figure 3). Nitrate to phosphate ratios in the inlet and coastal environments co-varied with a ratio of about 12:1, higher than the average elemental N:P stoichiometry of 5:1 for viral particles, but lower than the ratio of 16:1 associated with phytoplankton in balanced growth or heterotrophic bacteria [29,31]. Nutrient concentrations also co-varied with depth, with surface samples generally being low in nutrients. Furthermore, coastal samples generally showed lower nitrate concentrations than inlet samples. The majority of samples had relatively low phosphate concentrations compared to nitrate concentrations. This trend was reversed in the hypoxic samples with nitrate and phosphate concentrations being negatively correlated. 

### 3.2. Explanatory Power of Single Variable Linear Models

**Figure 4:**
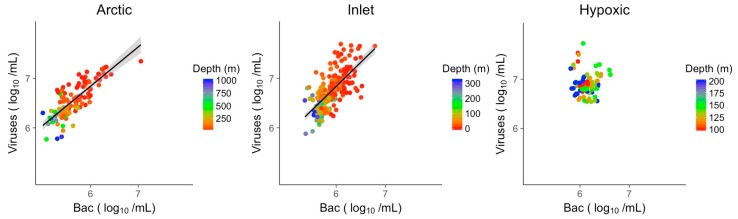
Linear models of log transformed viral abundances to log transformed bacterial abundances. Grey shading indicates the 95% confidence interval. Bac: Bacteria.

Linear models (LM) showing the distribution of direct relationships of log_10_ transformed viral abundances vs. log_10_ transformed bacterial abundances for the Arctic, inlet and hypoxic data sets are shown in Figure 4. For the inlet and Arctic data sets there were significant positive relationships between viral and bacterial abundances, explaining 48% of the variation in viral abundance in the inlet and 66% in the Arctic (Table 2). In the hypoxic samples, there was no discernable relationship between viral and bacterial abundances.

**Figure 5:**
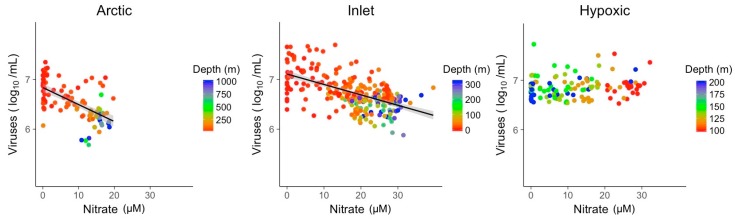
Linear models of log transformed viral abundance vs. nitrate (µM) concentration. Grey shading indicates the 95% confidence interval.

**Figure 6:**
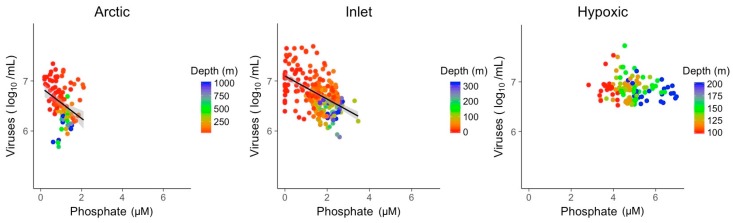
Linear models of log transformed viral abundance to phosphate (µM) concentration. Grey shading indicates the 95% confidence interval.

Nitrate and phosphate concentrations showed significant relationships with viral abundances in Arctic and inlet environments (Figure 5 and Figure 6). However, these relationships varied in strength and only explained ~10 to 40% of the variation in viral abundances (Table 2). For nitrate, the R^2^ values were 0.37 for Arctic samples and 0.33 for inlet samples, while for phosphate the values were 0.12 and 0.28, respectively. Relationships between viral abundances and nitrate or phosphate for the hypoxic samples were not significant. Generally, viral abundance and bacterial abundance were inversely correlated to depth, while nitrate and phosphate showed an opposite trend. However, this is not the case for the hypoxic samples. Based on the Shapiro–Wilk test, the residuals of the bivariate linear models were not normally distributed; however, the models displayed homogeneity of variance and the normal distribution of residuals, appropriate for large data sets (Figures S4–S9).

### 3.3. Multivariate Models Show Increased Explanatory Power

**Figure 7:**
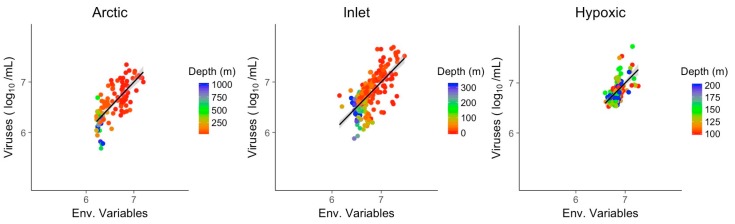
Generalized linear models of viral abundance and modeled abundance based on environmental variables for the Arctic, inlet and hypoxic environments, grey shading indicates the 95% confidence interval. Env.: Environmental variables.

Multivariate models of viral abundance were based on GLM of transformed data. For each environment, the best model was selected based on the AIC, and collinear predictors were reduced to one representative predictor. Combining only environmental variables and excluding bacterial abundance produced meaningful models in all three environments, matching or surpassing the explanatory power of bacterial abundance alone (Figure 7). The coefficient of determination for the three multivariate models was assessed by McFadden pseudo R^2^. Pseudo R^2^ of the GLMs and viral abundance in Artic, inlet and hypoxic environment were 0.56, 0.47 and 0.31, respectively. Significant predictors across all three environments were temperature and one of the nutrients (Table 3). Chlorophyll was a significant variable for the Arctic and hypoxic environments. Notably, for the inlet and hypoxic samples the models using combined environmental variables had an explanatory power that matched or exceeded the models based on bacteria only.

**Figure 8:**
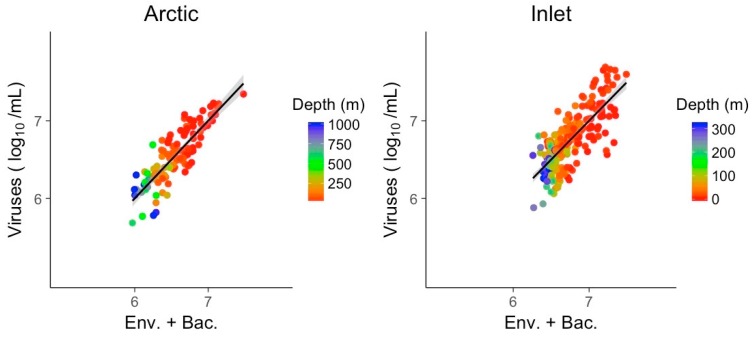
Generalized linear models of viral abundance and modeled abundance based on log-transformed bacterial abundances combined with environmental variables for the Arctic and inlet environments, grey shading indicates the 95% confidence interval. The model for the hypoxic environment did not improve by adding bacterial abundance relative to using environmental variables only in the model, and is not shown. Env.: Environmental variables; Bac.: Bacterial abundance.

The combined models of bacterial abundance and environmental variables substantially improved the relationship relative to bacterial abundances alone, for the Arctic and inlet environments (Figure 8). For the Arctic and inlet samples, pseudo R^2^ values were high, at 0.73 and 0.59, respectively. Again, best models were identified by the AIC for each environment and only one representative of collinear predictors was retained. Besides bacterial abundance, the only significant predictor in the models for both environments was nitrate (Table 4). Chlorophyll was a significant explanatory variable for the Arctic samples, while temperature was only significant for the inlet samples. For the hypoxic samples, including bacterial abundance did not significantly improve the explanatory power of the combined environmental variables over viral abundance, and was left out.

Using environmental variables, the improvement over models solely based on bacterial abundances was stronger for the inlet and hypoxic samples than for Arctic samples. GLMs for samples where PAR data were available showed that PAR was not a significant predictor and did not improve the explanatory power of the models. Additionally, based on the Shapiro–Wilk test, the residuals for the GLMs were not normally distributed. However, the residuals were centered around zero and the deviation from the normal distribution appeared random; all GLMs demonstrated homogeneity of variance to a level that can be expected for models of this size (Figures S10–S13). While other model approaches on these data sets produced higher explanatory power, this came at the expense of more pronounced heterogeneity of variance.

## 4. Discussion

As has been found in many previous studies, viruses are typically about ten times more abundant than bacteria in marine surface waters, although there is wide variation around this mean across environments [10,12] that is difficult to explain [11]. In this study, we used a series of models of varying complexity to investigate relationships between viral abundances and several environmental variables in an effort to explain the factors responsible for variation in viral abundances. We found that viral abundances across locations and time were related to a suite of environmental factors, but particularly nitrogen and phosphorus concentrations, as well as bacterial abundances. The exception was hypoxic environments, in which viral abundances were only explained by a combination of physical and chemical factors. These findings are discussed in detail below.

A database was compiled from samples collected from different depths, across a wide geographic range at different times of year. The values of environmental variables, including bacterial and viral abundances, were in the typical ranges for these habitats. One set of outlying data from Rivers Inlet was excluded from the models because of excessively high viral and bacterial abundances that could not be related to any of the environmental variables or explained in a model. Presumably, these data were due to high rates of bacterial growth and a lysis event during sampling, and show the difficulty in accounting for such extremes in models.

Samples were classified into Arctic, inlet and hypoxic environments. The LDA of the environmental variables for the three environments supported the approach to classifying samples based on the prevailing conditions, rather than by geographic location, cruise or project. The Arctic and inlet samples represent a continuum of environmental conditions. In contrast, the hypoxic samples were collected from depths below 100 m, had dissolved oxygen concentrations below 1.5 mL·L^−1^ and an altered nitrate to phosphate stoichiometry; thus, they represent a much different environment [44,48]. 

Given the stoichiometry of viral particles, nitrogen and phosphorus are key resources for viral replication and their availability would be expected to affect viral production. Nitrate to phosphate ratios averaged about 12:1 for the Arctic and inlet data, although in some cases reached much higher values for the inlet samples. This ratio was higher than the estimated elemental ratio of 5:1 for viral particles [29], but lower than the nitrate to phosphate ratio of ~15:1 previously found in marine samples [49]. The ratio of nitrate to phosphate was inverted to 1:12 in the hypoxic samples, as nitrate is used as an alternative electron acceptor by bacteria under anoxic conditions [48,50]. Arctic surface and hypoxic deep samples display the potential for nitrate limitation during viral replication in some virus–host systems with concentrations approaching zero. Nitrate and phosphate ratios in seawater show a similarity to the elemental nitrogen and phosphorus ratios in cells [31,51]. Consequently, shifts in the nitrate to phosphate ratio in seawater could link to the nitrogen and phosphorus supply to cells. When growing at relatively low phosphate concentrations, the high phosphorus accumulation of up to 87% of the cellular content in viral particles [29] could lead to a limitation in phosphorus supply during viral replication in autotrophic hosts.

The strength of relationships between viral abundance and single variables differed among the subsets of data. The explanatory power of bacterial abundance was higher for the Arctic data (R^2^ = 0.66) than for the inlets data (R^2^ = 0.48), although both were comparable to relationships reported for other surface and sub-surface studies [9,10]. Relationships of viral abundances to nitrate or phosphate were weaker than for bacterial abundance in the Arctic and inlet samples; however, the significant explanatory power of nitrate (R^2^ = 0.37 and 0.33) in the Arctic and inlet environments comes close to that of bacterial abundance, highlighting the importance of nitrate. In the Arctic and inlet models, viral abundance and depth covaried; however, within the scope of this study, we treated depth as a co-variate for the environmental variables, e.g., salinity, temperature or light, rather than as an independent variable. That viral abundance was not significantly related to any of the three single variables in the hypoxic data implies that viral production is dependent on different processes in this environment.

Combining environmental variables into multivariate models showed high explanatory power of viral abundance in all environments. Based on the pseudo R^2^ values, the models for the Arctic and the inlet data explained about 50% of the variation in viral abundance; for the inlet data, environmental variables surpassed the explanatory power of bacterial abundance alone. For the hypoxic data, the explanatory power of environmental variables was 31%, a substantial improvement compared to the absence of significant correlations with bacterial abundance, nitrate, or phosphate alone. After removing collinear nutrient variables, significant components of the models across data sets were temperature, chlorophyll and representative nutrients, nitrate, phosphate and silicate. 

Phosphate was a significant component of the model for the hypoxic environment, but not for the Arctic or inlet samples, which generally had higher nitrate to phosphate ratios than the hypoxic samples. Phosphate is important to viral replication and infection, highlighted by reduced viral mortality of phytoplankton under phosphate limitation [33]. However, the collinearity of nitrate and phosphate data in the Arctic and inlet samples makes it difficult to identify which nutrient is eventually affecting viral replication. That phosphate was a statistically more significant variable than nitrate in the hypoxic model is presumably a result of the full depletion of nitrate by denitrification in samples that are truly anoxic [48,50]. 

The observation that chlorophyll was a significant variable in the Arctic but not in the inlet samples can be explained by phytoplankton blooms in the Arctic, which are associated with increases in viral abundance. For example, a seasonal study in the Beaufort Sea shelf showed a significant correlation between chlorophyll and viral abundance [52], as did another study in fresh waters [53]. The significance of chlorophyll in the deep hypoxic environment, however, must be related to phytoplankton cells sinking out of the photic zone, or is a statistical artefact. Based on the data presented, using chlorophyll as a proxy indicates that phytoplankton were not important in the inlet environments, where the majority of viruses are produced by and infect heterotrophic bacteria. Overall, it is remarkable that multivariate models built from environmental variables alone explain viral abundance as well as, or even exceed, the explanatory power of bacterial abundance.

Combining data for environmental variables and bacterial abundance further improved the explanatory power of the models for the Arctic and inlet data, with 73 and 59% of the variation in viral abundance explained by the multivariate models. In contrast, for the hypoxic data, including bacterial abundance did not increase the explanatory power from the multivariate model using environmental variables only. This suggests a strong effect on viral production by nutrient stoichiometry and other environmental conditions. Across these multivariate models, the consistent component besides bacterial abundance was nitrate. While temperature or salinity were significant variables in the models for the Arctic and inlet environments, again, chlorophyll was only a significant variable in the Arctic environment and can be explained by phytoplankton blooms [52,53]. The influence of environmental variables on the relationship between viral and bacterial abundances, and the differences among environments, is consistent with observations from marine and freshwater environments [10,12]. The data presented here show that much of this variation is likely explained by differences in nutrient availability.

In conclusion, the environmental variables examined here are associated with changes in viral abundance and the relationship between viruses and bacteria in diverse marine samples. We provide a first attempt at generalized statistical models that capture these relationships, and a first step towards a better ecological understanding of the processes controlling virus abundance in the ocean. For the purpose of explanatory models, samples can be classified by their environment, rather than arbitrarily by project, cruise or station. While bacterial abundance is a well-established predictor for viral abundance, it fails in certain marine environments, and can be substantially improved by more complex models incorporating environmental variables. Individual environmental variables do not have great explanatory power for predicting viral abundances; yet, when combined in multivariate models they can produce explanatory power equal to or surpassing that of bacterial abundance. This study shows that the environmental variables explaining viral abundance vary among environments, but nutrient concentrations, as well as salinity and temperature, appear to be key factors. The relationships described here only apply to viruses that can be detected by flow cytometry. RNA viruses with small genomes can be difficult to detect and distinguish by flow cytometry and may have different relationships to environmental variables.

The three types of environments studied in this project are predicted to be strongly affected by climate change, with increased stratification in inlets, the North Atlantic, Arctic and Northeast Pacific, and associated changes in vertical nutrient fluxes and expanding oxygen minimum zones [54,55,56,57]. Understanding the interplay between viruses, hosts and environmental variables in these types of environments improves the potential of predicting how virus-host systems will respond to environmental changes.

## Figures and Tables

**Table 1:** Ranges, mean values and units of data included in the statistical analysis. PAR: Photosynthetically active radiation; PSU: Practical salinity units.

Variable	Min.	Max.	Mean	Unit
**Temperature**	−1.710	15	7	°C
**Salinity**	3.060	35	31	PSU
**Chlorophyll**	0.030	44	2	mg·m^−3^
**Oxygen**	0.005	10	4	mL·L^−1^
**PAR**	0.000	669	25	µmol quanta m^−2^·s^−1^
**NO_3_**	0.010	54	15	µM
**PO_4_**	0.006	7	2	µM
**SiO_4_**	0.070	141	43	µM
**Bacteria**	7.31 × 10^4^	7.40 × 10^7^	1.66 × 10^6^	Cells mL^−1^
**Viruses**	4.83 × 10^5^	1.40 × 10^8^	8.35 × 10^6^	Viruses mL^−1^

**Table 2:** Results for the significant linear models of viral abundance and bacterial abundance, nitrate and phosphate in the Arctic and inlet environments. Samples from the hypoxic environment did not show significant relationships and are not listed.

Variable	Parameter	Arctic	Inlet
**Bacteria (log_10_)**	**R^2^**	0.66	0.48
	**Slope**	0.80	0.97
	***p*-value**	2.5 × 10^−27^	1.9 × 10^−37^
**NO_3_**	**R^2^**	0.37	0.33
	**Slope**	−0.03	−0.02
	***p*-value**	1.4 × 10^−12^	1.2 × 10^−24^
**PO_4_**	**R^2^**	0.12	0.28
	**Slope**	−0.31	−0.23
	***p*-value**	1.0 × 10^−04^	3.6 × 10^−20^

**Table 3:** Results and significant predictors of generalized linear models based on environmental variables (Env.) per environment. Akaike Information Criterion (AIC), pseudo R^2^, sample size (*n*) and degrees of freedom (df) shown with the effect sizes for predictors, fonts indicate the significance level.

Env.	Arctic	Inlet	Hypoxic
McFadden (R^2^)	0.56	0.47	0.31
Slope	1.00	1.00	1.00
*n*/df	109/104	261/258	126/122
Intercept	5.545	3.75	13.721
Temperature	0.141	1.068	−2.763
Salinity	-	0.199	-
Chlorophyll	0.191	-	0.384
Oxygen	0.112	-	-
NO_3_	−0.018	−0.013	-
PO_4_	-	-	−0.078
SiO_4_	0.009	-	0.004
PAR	-	-	-
Signif. level	<0.01	<0.05	*<0.1*

**Table 4:** Results and significant predictors of combined generalized linear models based on environmental variables and bacterial abundance (Env. + Bac.) for the Arctic and inlet environment. AIC, pseudo R^2^, sample size (*n*) and degrees of freedom (df) shown with the effect sizes for predictors, fonts indicate the significance level.

Env. + Bac.	Arctic		Inlet	
McFadden (R^2^)	0.73		0.59	
Slope	1.00		1.00	
n/df	109/105		252/249	
Intercept	5.008		1.020	
Temperature	-		0.774	
Salinity	*−0.523*		-	
Chlorophyll	0.098		-	
Oxygen	-		-	
NO_3_	−0.010		*−0.003*	
PO_4_	-		-	
SiO_4_	-		-	
PAR	-		-	
Bacteria (log_10_)	0.607		0.665	
Signif. level	<0.01	<0.05		*<0.1*

## Supplementary Materials

The following are available online at www.mdpi.com/1999-4915/9/6/152/s1, Table S1: Viral, bacterial and environmental data used in building the models. Figure S1: Data distribution and direct correlations (Pearson’s) of data in the Arctic environment, Figure S2: Data distribution and direct correlations (Pearson’s) of data in the inlet environment, Figure S3: Data distribution and direct correlations (Pearson’s) of data in the hypoxic environment, Figure S4: Residual density for linear models of log_10_ viral abundance and log_10_ bacterial abundance in the three environments. Shapiro–Wilk test: Arctic, w = 0.99, *p*-value = 0.54; inlet, w = 0.96, *p*-value = 0.0002; hypoxic, 0.93, *p*-value = 0.0001, Figure S5: Residual distribution and qq-plots for linear models of log_10_ viral abundance and log_10_ bacterial abundance for the Arctic (a), inlet (b) and hypoxic (c) environments, Figure S6: Residual density for linear models of log_10_ viral abundance and nitrate in the three environments. Shapiro–Wilk test: Arctic, w = 0.98, *p*-value = 0.087; inlet, w = 0.99, *p*-value = 0.176; hypoxic, 0.93, *p*-value = 3.61 × 10^−6^, Figure S7: Residual distribution and qq-plots for linear models of log_10_ viral abundance and nitrate for the Arctic (a), inlet (b) and hypoxic (c) environments, Figure S8: Residual density for linear models of log_10_ viral abundance and phosphate in the three environments. Shapiro–Wilk test: Arctic, w = 0.97, *p*-value = 0.031; inlet, w = 0.99, *p*-value = 0.034; hypoxic, 0.93, *p*-value = 7.69 × 10^−6^, Figure S9: Residual distribution and qq-plots for linear models of log_10_ viral abundance and phosphate for the Arctic (a), inlet (b) and hypoxic (c) environments, Figure S10: Residual density for generalized linear models of log_10_ viral abundance and combined environmental variables in the three environments. Shapiro–Wilk test: Arctic, w = 0.99, *p*-value = 0.406; inlet, w = 0.95, *p*-value = 1.68 × 10^−7^; hypoxic, 0.95, *p*-value = 6.24 × 10^−5^, Figure S11: Residual distribution and qq-plots for linear models of log_10_ viral abundance and combined environmental variables for the Arctic (a), inlet (b) and hypoxic (c) environments, Figure S12: Residual density for generalized linear models of log_10_ viral abundance and combined log_10_ bacterial abundance and environmental variables in the Arctic and inlet environments. Shapiro–Wilk test: Arctic, w = 0.99, *p*-value = 0.685; inlet, w = 0.98, *p*-value = 0.002, Figure S13: Residual distribution and qq-plots for linear models of log_10_ viral abundance and combined log_10_ bacterial abundance and environmental variables for the Arctic (a) and inlet (b) environments.
