# Supplementary material for: Nutrients and Other Environmental Factors Influence Virus Abundances across Oxic and Hypoxic Marine Environments
Source: Viruses. 2017 Jun 17;9(6):152. doi: 10.3390/v9060152 (PMC5490827; doi:10.3390/v9060152)
Supplement: Supplementary file 1 [file viruses-09-00152-s001.zip › viruses-187399_2proofreading_supplementary/viruses-187399_2proofreading_supplementary.docx]

Supplementary Materials: Nutrients and Other Environmental Factors Influence Virus Abundances across Oxic and Hypoxic Marine Environments

­


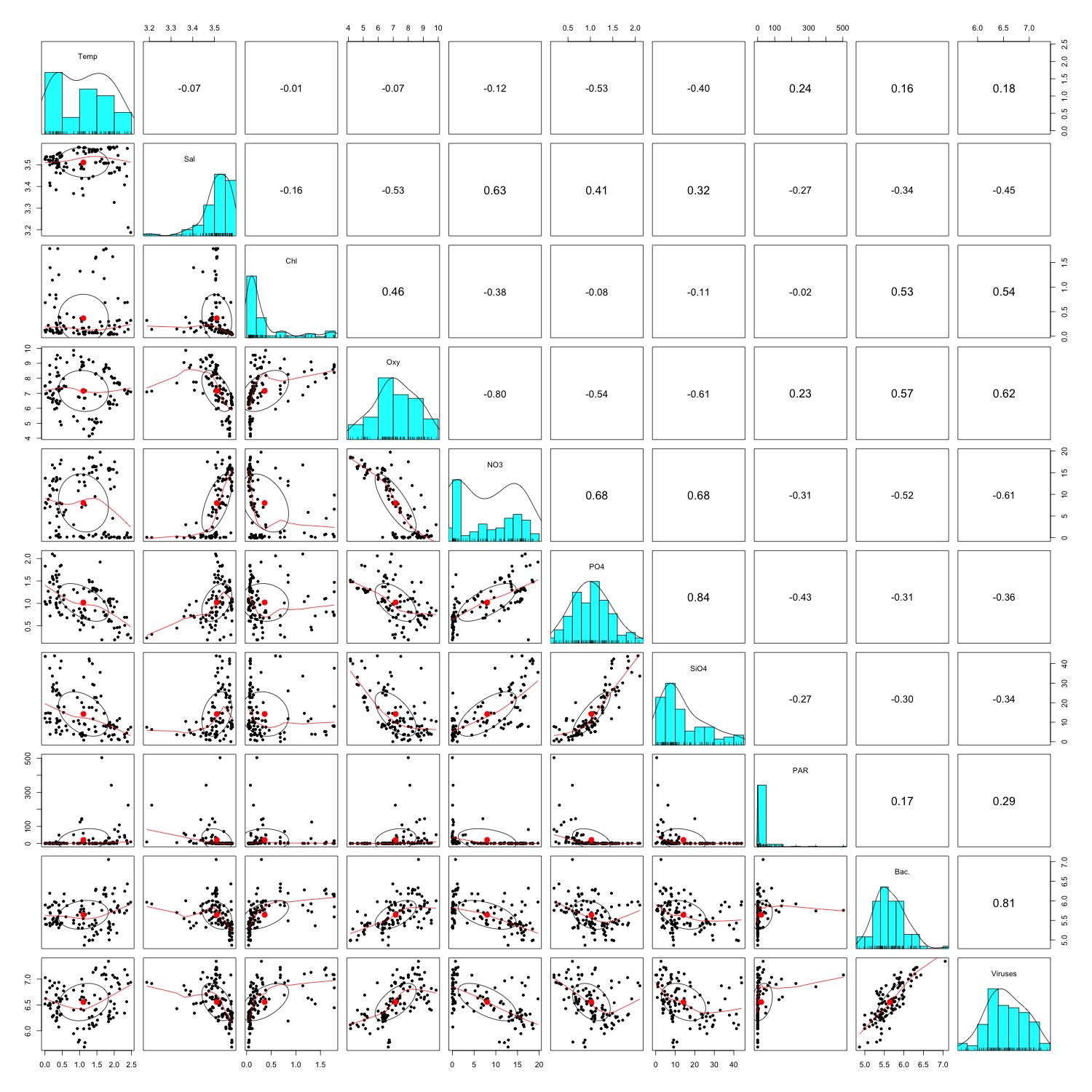


**Figure S1.** Data distribution and direct correlations (Pearson’s) of data in the Arctic environment.


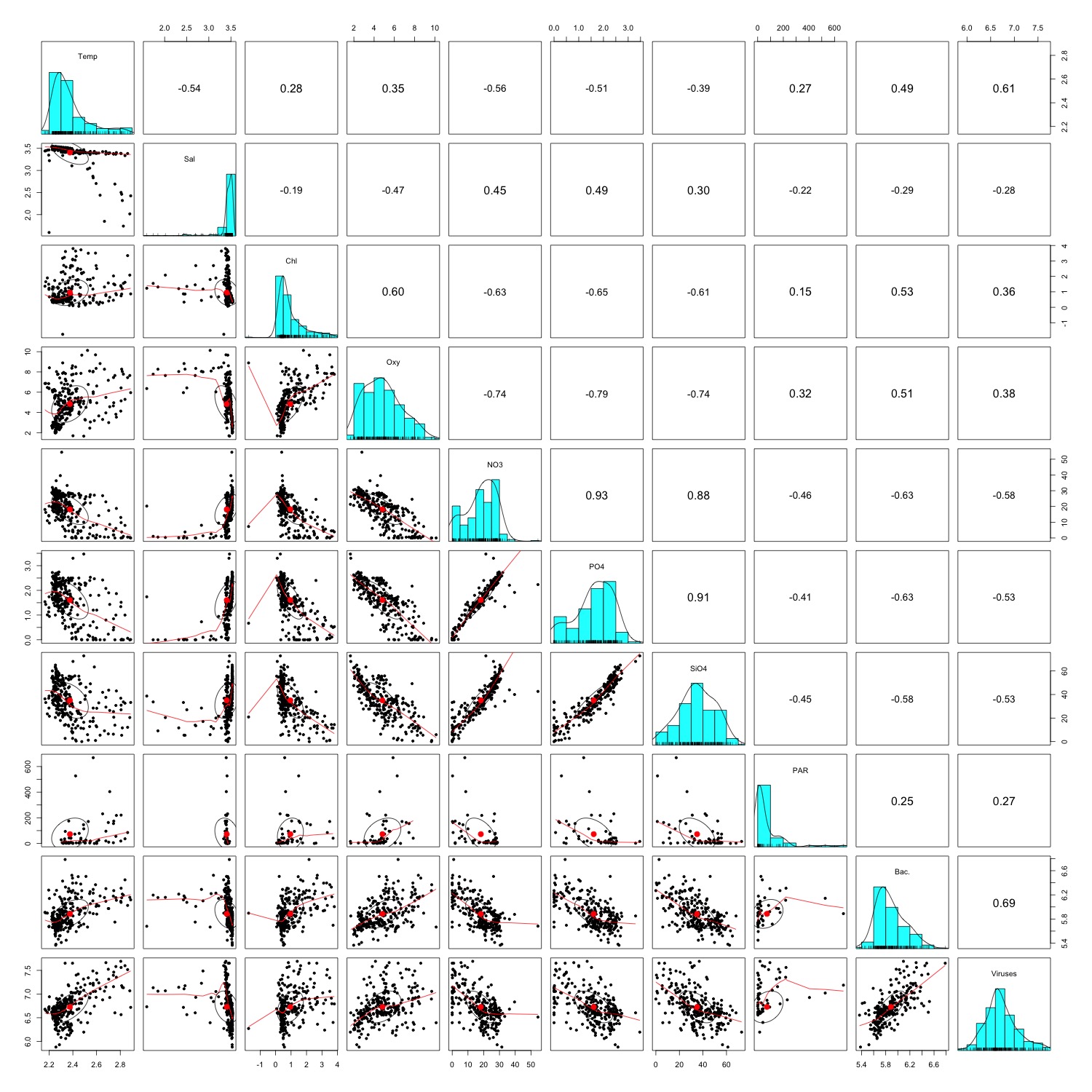


**Figure S2.** Data distribution and direct correlations (Pearson’s) of data in the inlet environment.


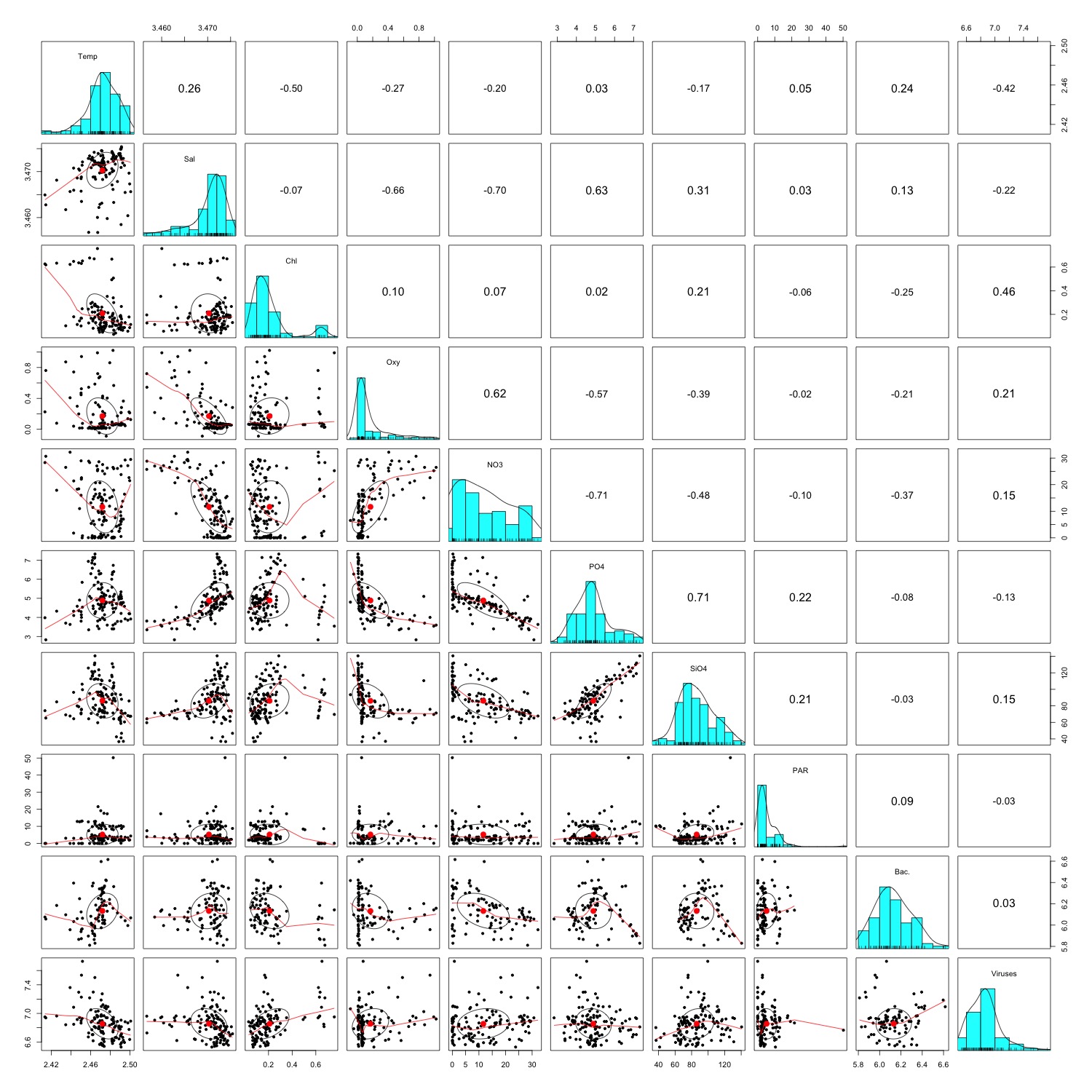


**Figure S3.** Data distribution and direct correlations (Pearson’s) of data in the hypoxic environment.


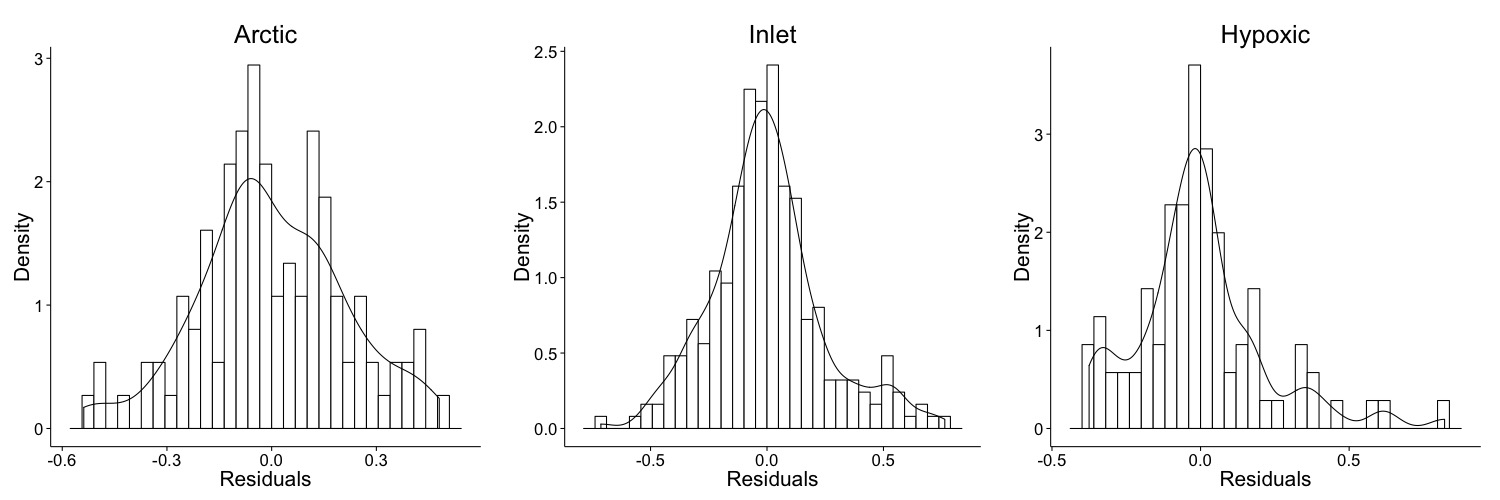


**Figure S4.** Residual density for linear models of log_10_ viral abundance and log_10_ bacterial abundance in the three environments. Shapiro–Wilk test: Arctic, w = 0.99, *p*-value = 0.54; inlet, w = 0.96, *p*-value = 0.0002; hypoxic, 0.93, *p*-value = 0.0001.

(a)


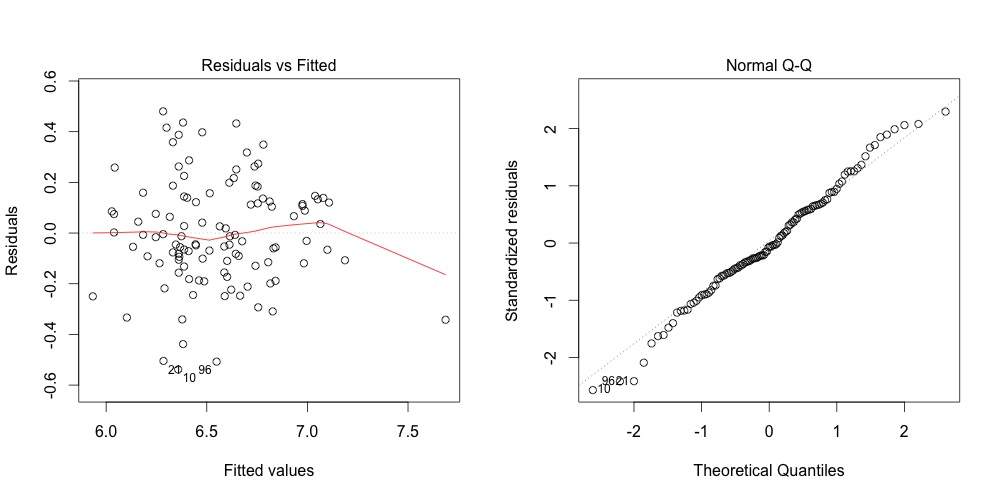


(b)


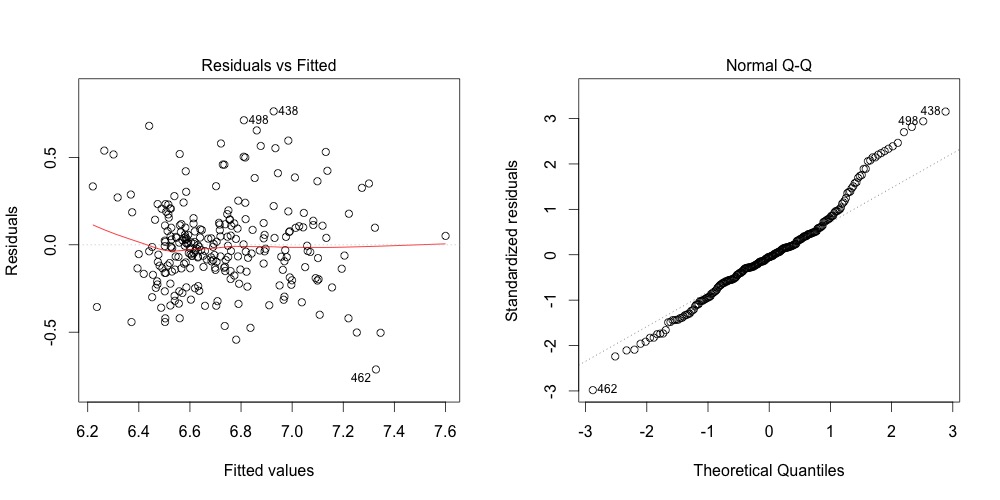


(c)


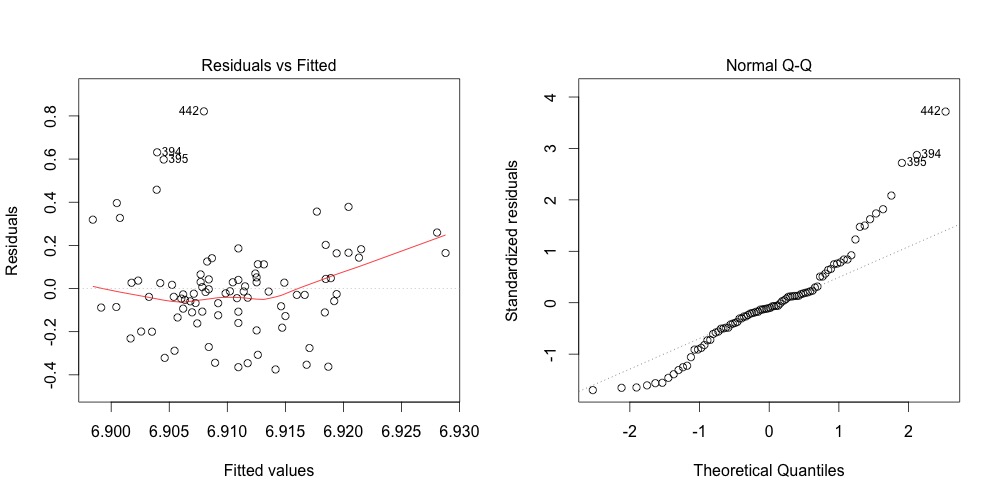


**Figure S5.** Residual distribution and qq-plots for linear models of log_10_ viral abundance and log_10_ bacterial abundance for the Arctic (**a**), inlet (**b**) and hypoxic (**c**) environments.


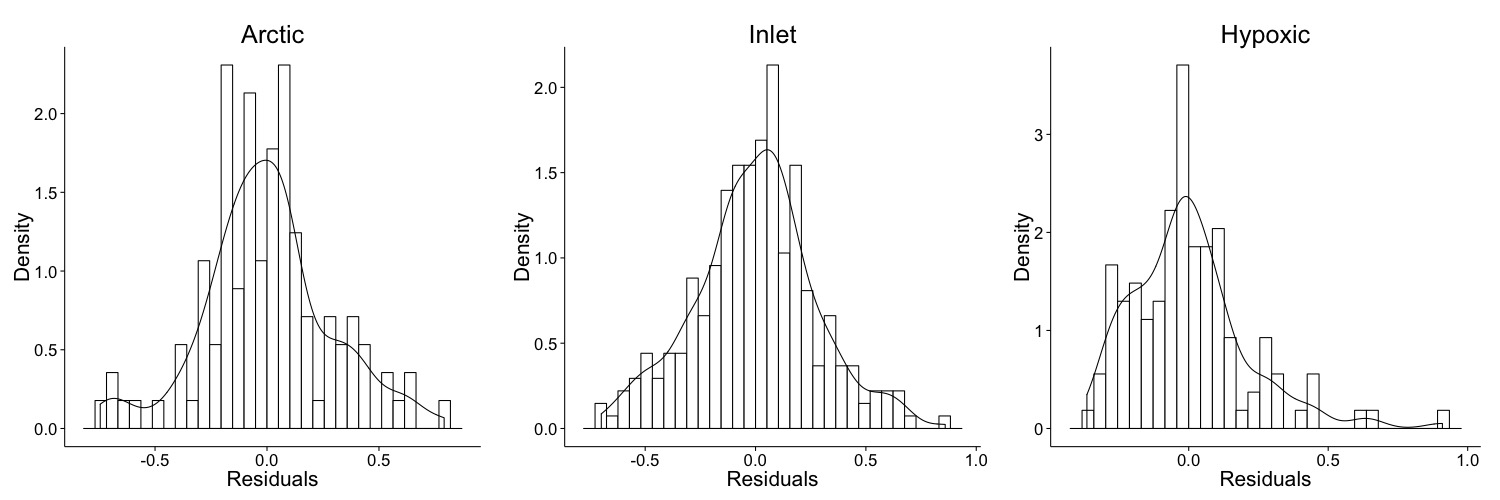


**Figure S6.** Residual density for linear models of log_10_ viral abundance and nitrate in the three environments. Shapiro–Wilk test: Arctic, w = 0.98, *p*-value = 0.087; inlet, w = 0.99, *p*-value = 0.176; hypoxic, 0.93, *p*-value = 3.61e-06.


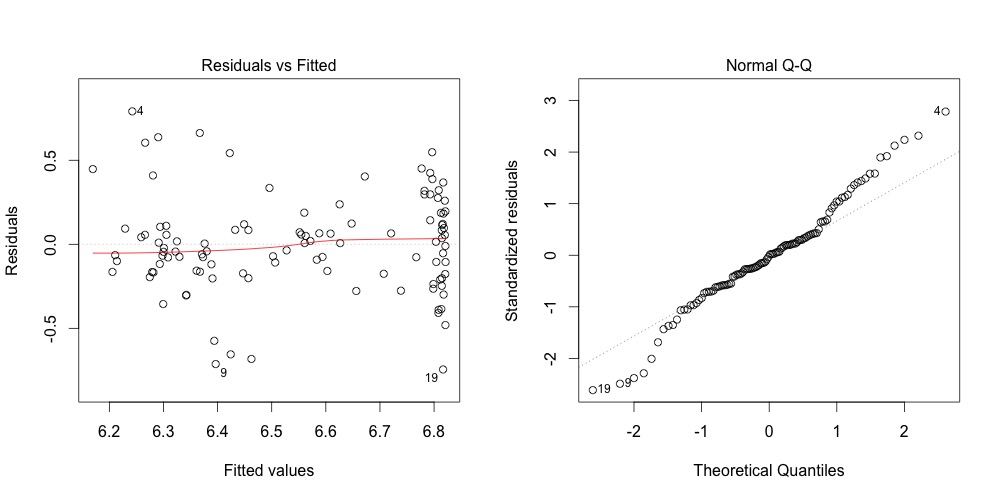


(b)

(a)


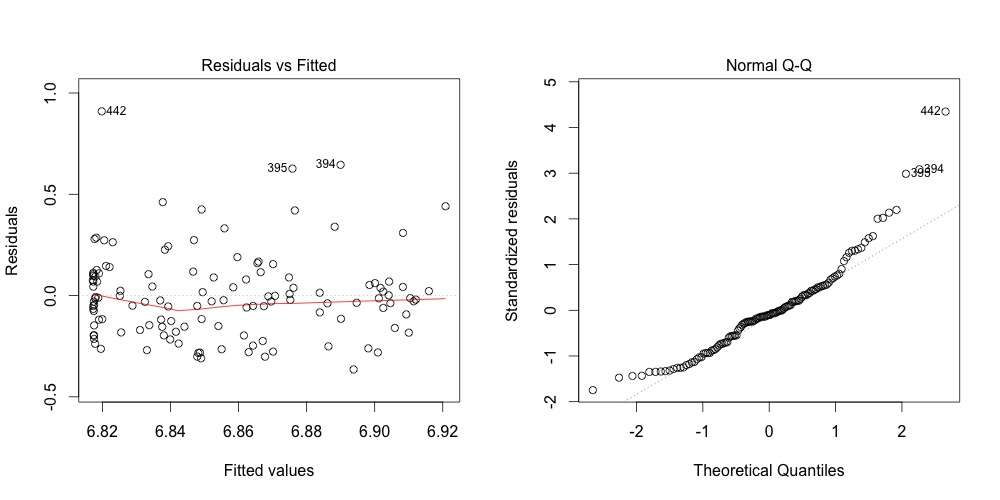

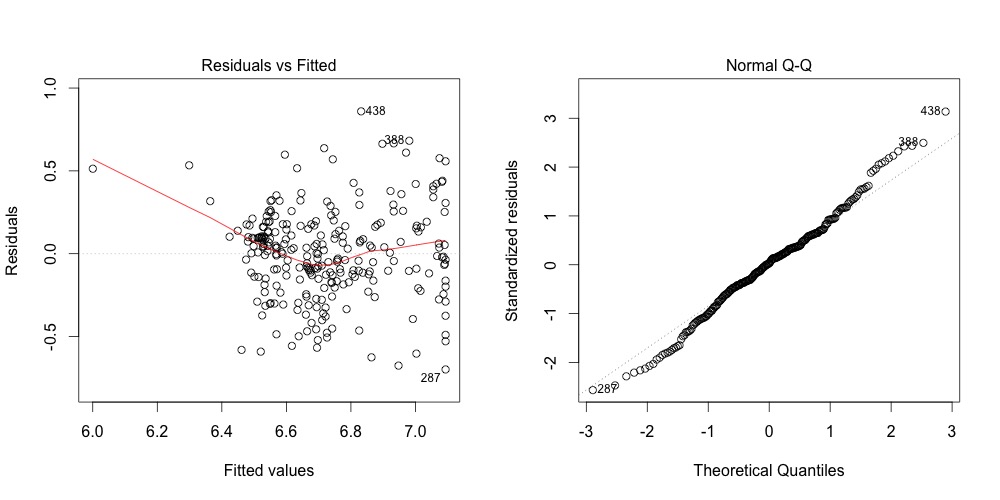


(c)

**Figure S7.** Residual distribution and qq-plots for linear models of log_10_ viral abundance and nitrate for the Arctic (**a**), inlet (**b**) and hypoxic (**c**) environments.


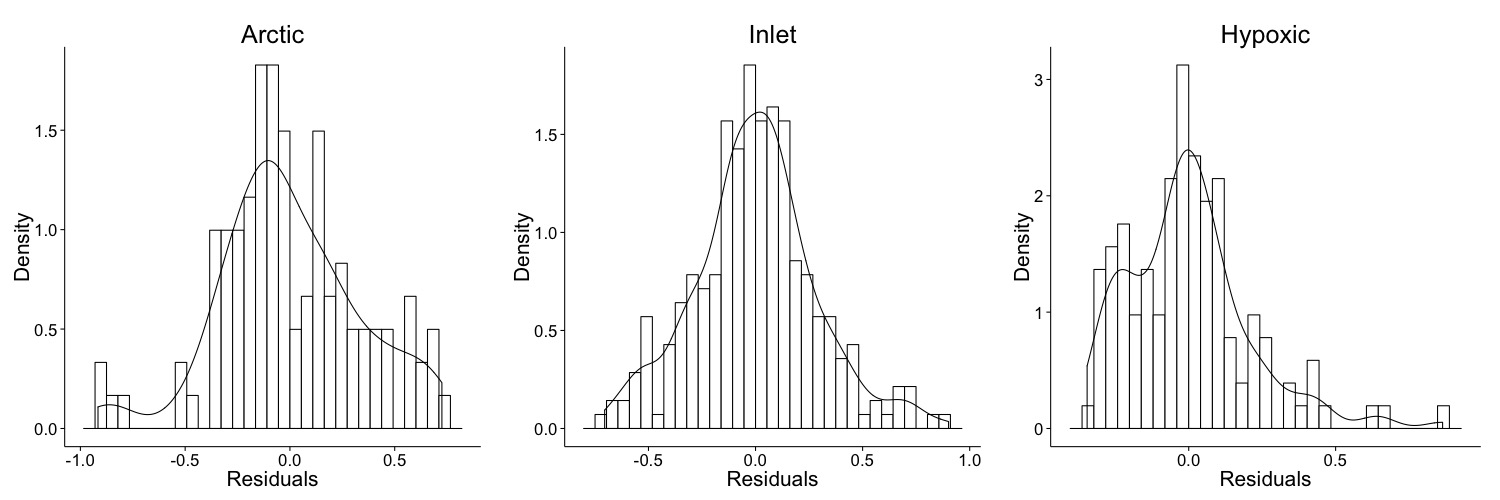


**Figure S8.** Residual density for linear models of log_10_ viral abundance and phosphate in the three environments. Shapiro–Wilk test: Arctic, w = 0.97, *p*-value = 0.031; inlet, w = 0.99, *p*-value = 0.034; hypoxic, 0.93, *p*-value = 7.69e-06.


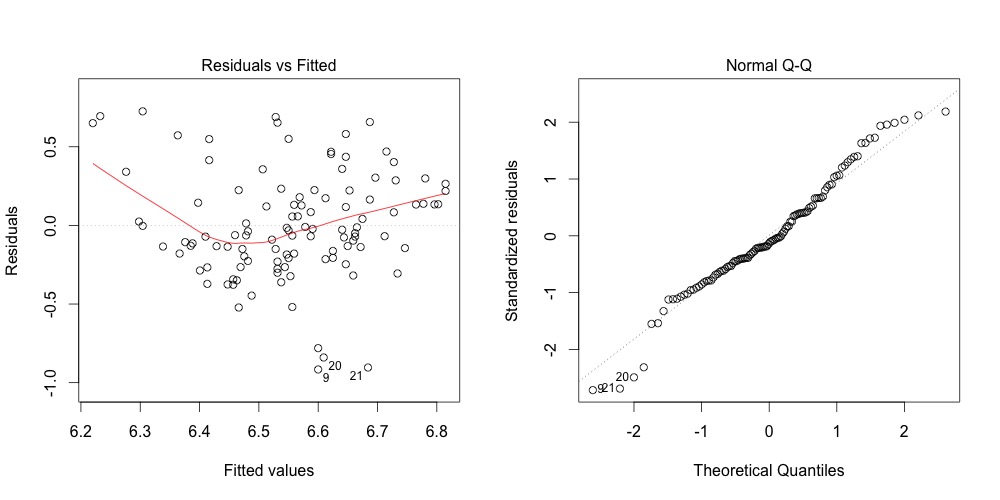


(c)

(b)

(a)


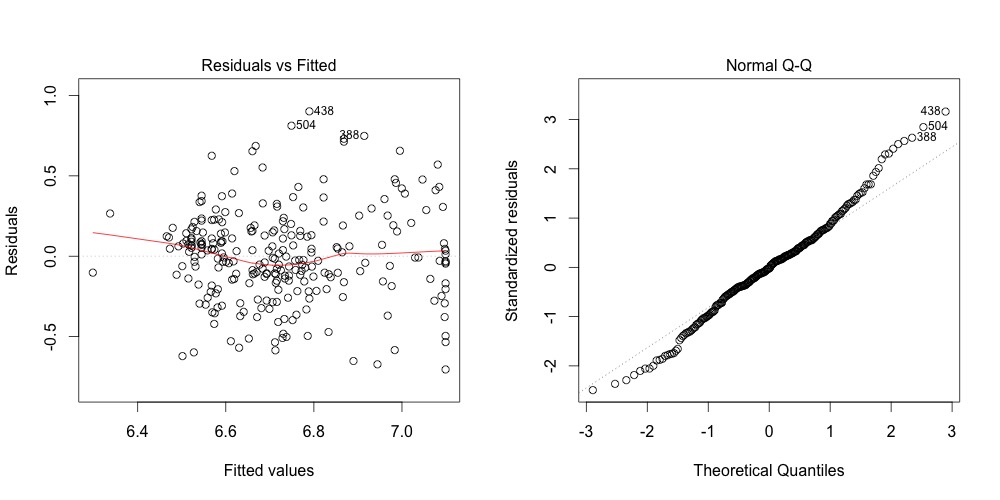


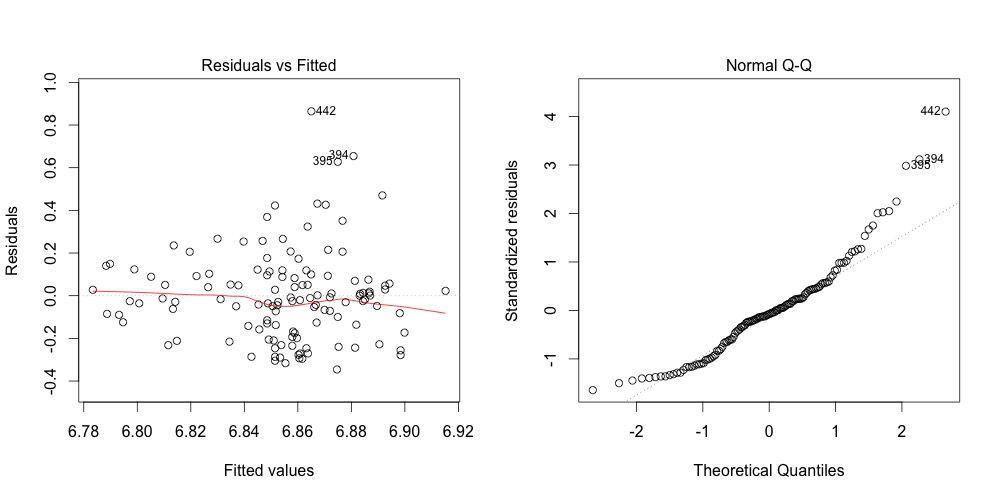


**Figure S9.** Residual distribution and qq-plots for linear models of log_10_ viral abundance and phosphate for the Arctic (**a**), inlet (**b**) and hypoxic (**c**) environments.


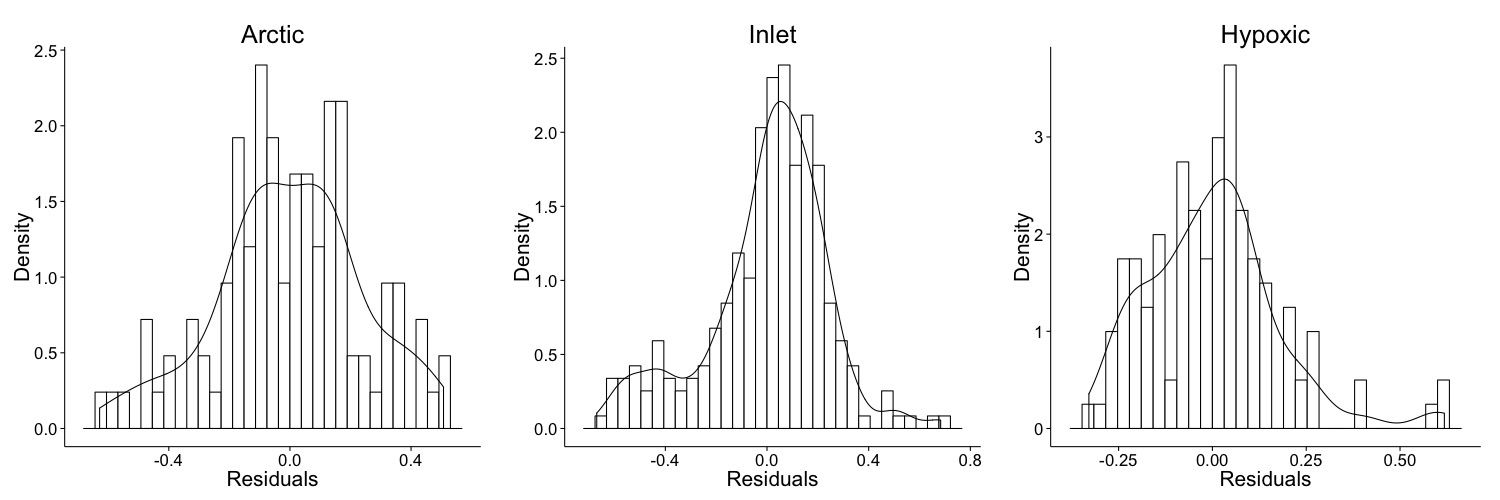


**Figure S10.** Residual density for generalized linear models of log_10_ viral abundance and combined environmental variables in the three environments. Shapiro–Wilk test: Arctic, w = 0.99, *p*-value = 0.406; inlet, w = 0.95, *p*-value = 1.68e-07; hypoxic, 0.95, *p*-value = 6.24e-05.


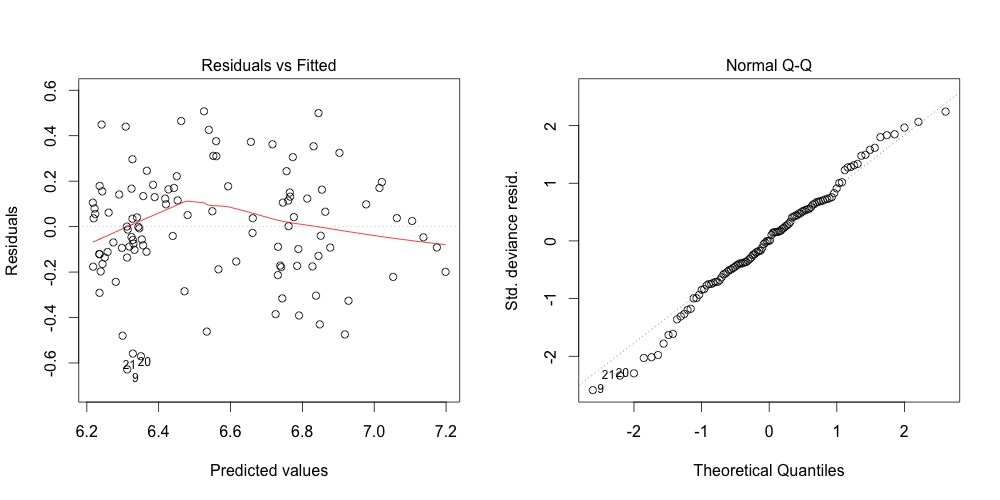


(b)

(a)


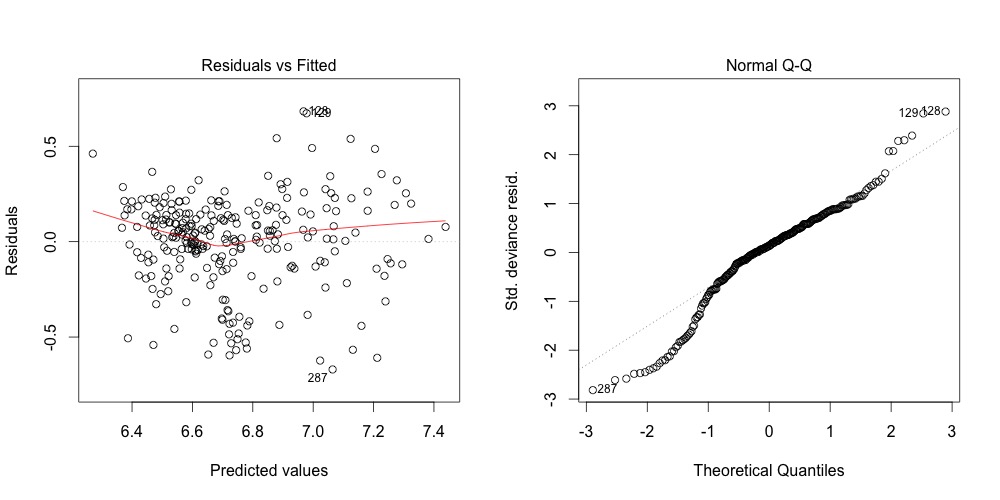


(c)


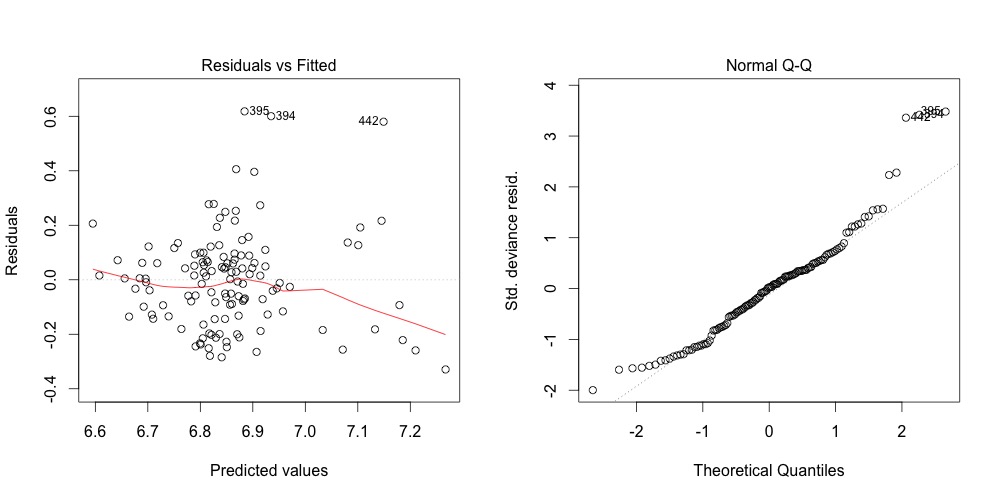


**Figure S11.** Residual distribution and qq-plots for linear models of log_10_ viral abundance and combined environmental variables for the Arctic (**a**), inlet (**b**) and hypoxic (**c**) environments.

**
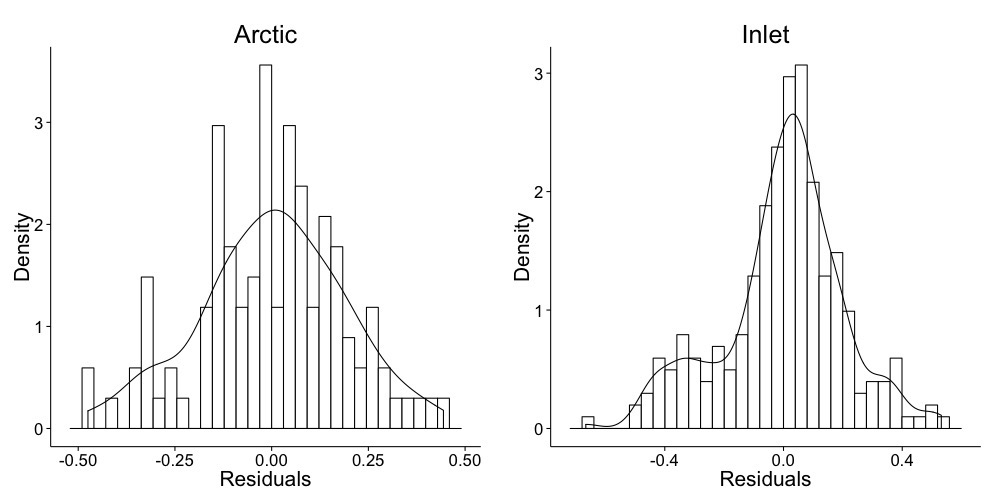
**

**Figure S12.** Residual density for generalized linear models of log_10_ viral abundance and combined log_10_ bacterial abundance and environmental variables in the Arctic and inlet environments. Shapiro–Wilk test: Arctic, w = 0.99, *p*-value = 0.685; inlet, w = 0.98, *p*-value = 0.002.


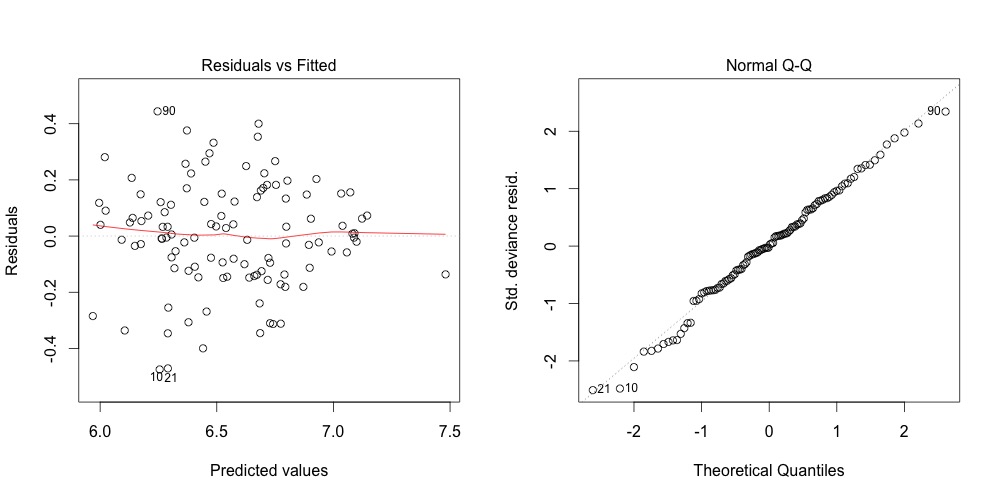


(a)

(b)


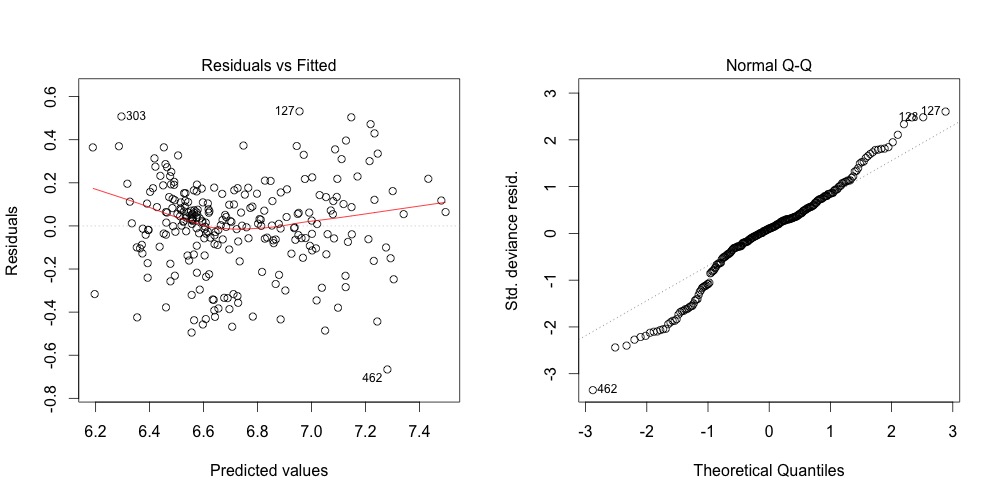


**Figure S13.** Residual distribution and qq-plots for linear models of log_10_ viral abundance and combined log_10_ bacterial abundance and environmental variables for the Arctic (**a**) and inlet (**b**) environments.
