# Supplementary figures and images for: Nutrients and Other Environmental Factors Influence Virus Abundances across Oxic and Hypoxic Marine Environments
Source: Viruses. 2017 Jun 17;9(6):152. doi: 10.3390/v9060152 (PMC5490827; doi:10.3390/v9060152)

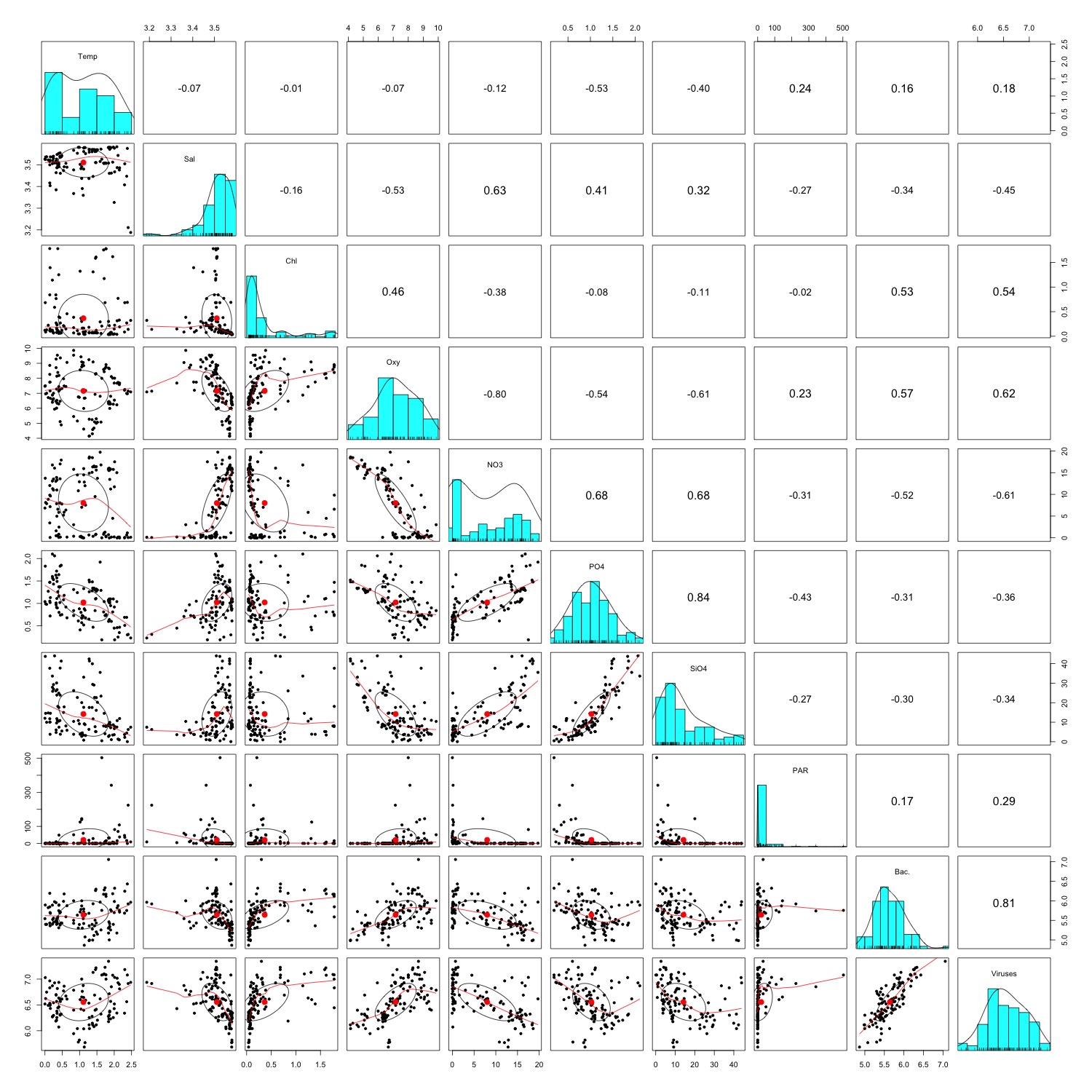

Supplement: Supplementary file 1 [file viruses-09-00152-s001.zip › viruses-187399_2proofreading_supplementary/FigureS1.jpeg]

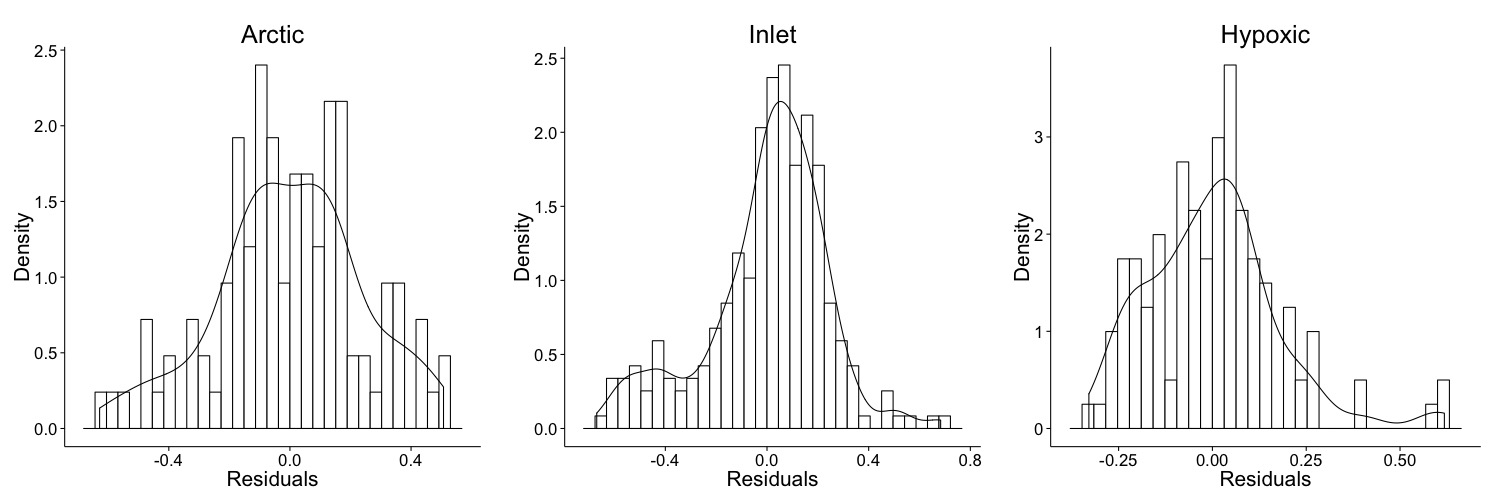

Supplement: Supplementary file 1 [file viruses-09-00152-s001.zip › viruses-187399_2proofreading_supplementary/FigureS10.jpeg]

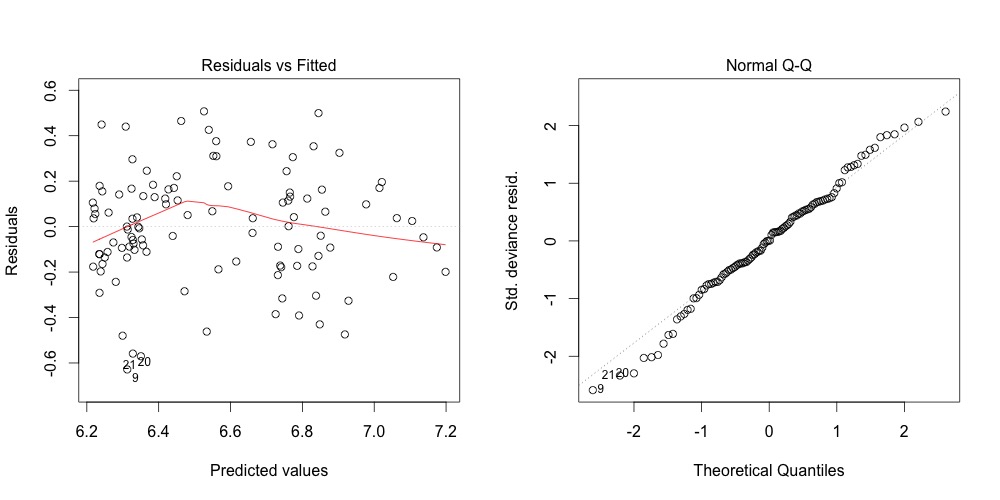

Supplement: Supplementary file 1 [file viruses-09-00152-s001.zip › viruses-187399_2proofreading_supplementary/FigureS11a.jpeg]

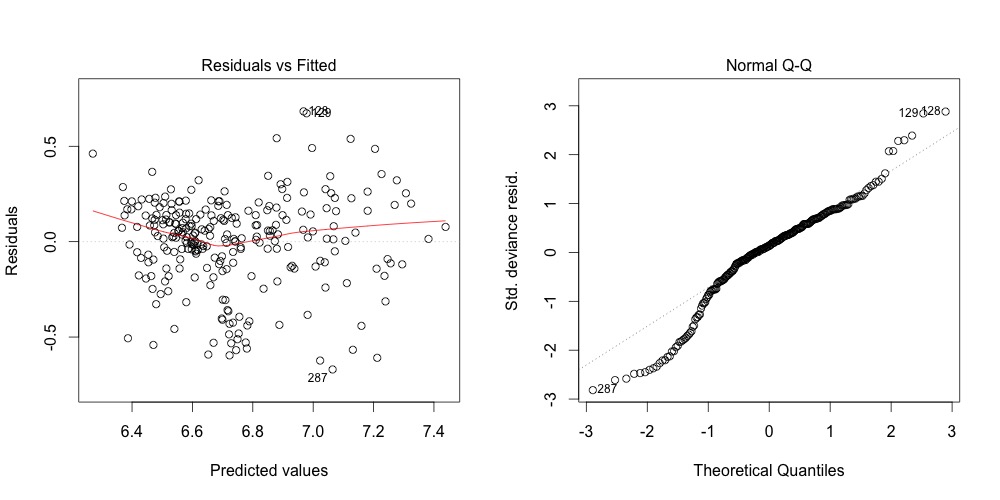

Supplement: Supplementary file 1 [file viruses-09-00152-s001.zip › viruses-187399_2proofreading_supplementary/FigureS11b.jpeg]

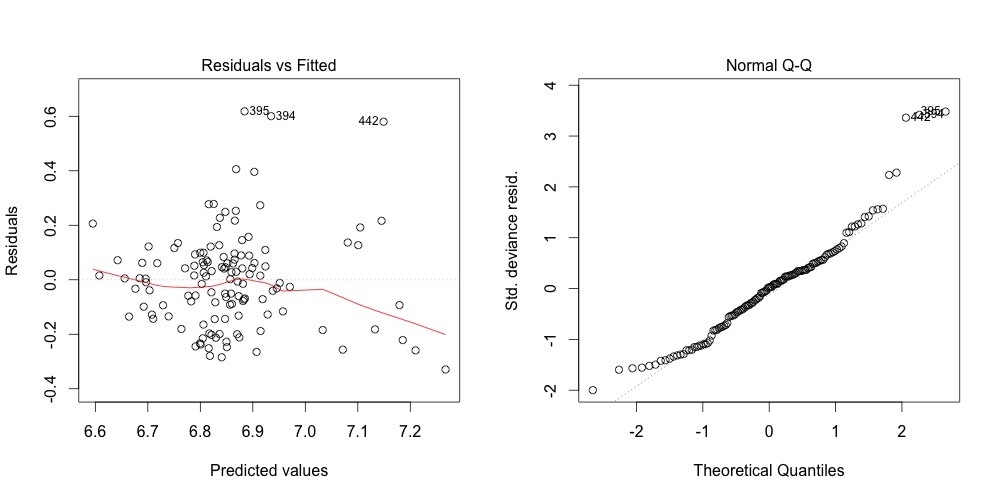

Supplement: Supplementary file 1 [file viruses-09-00152-s001.zip › viruses-187399_2proofreading_supplementary/FigureS11c.jpeg]

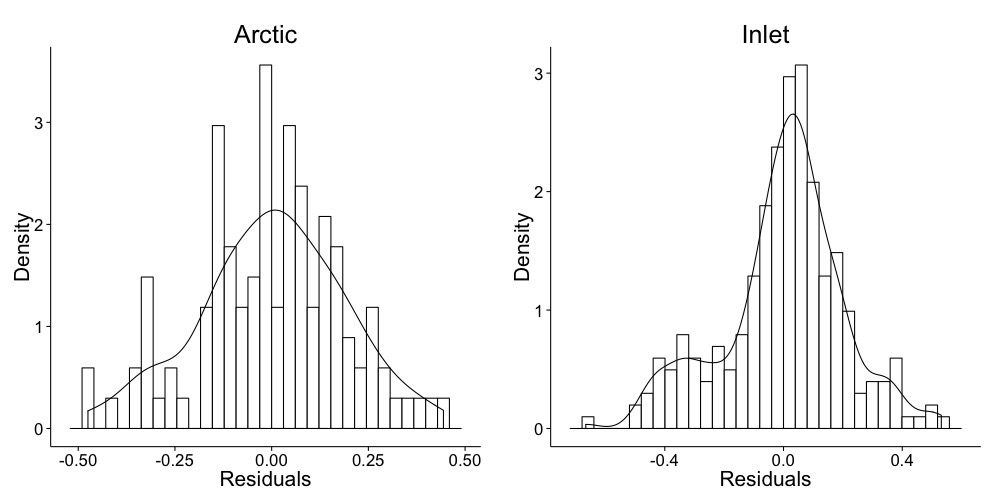

Supplement: Supplementary file 1 [file viruses-09-00152-s001.zip › viruses-187399_2proofreading_supplementary/FigureS12.jpeg]

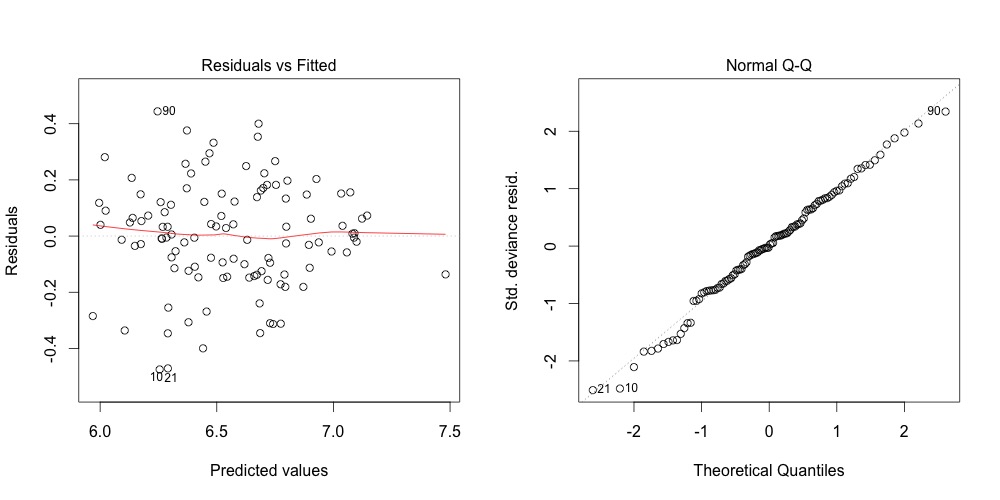

Supplement: Supplementary file 1 [file viruses-09-00152-s001.zip › viruses-187399_2proofreading_supplementary/FigureS13a.jpeg]

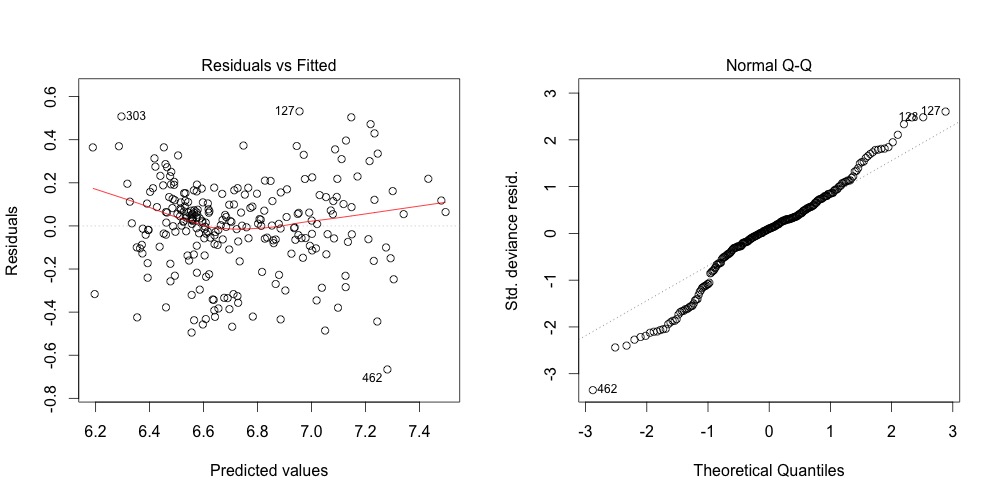

Supplement: Supplementary file 1 [file viruses-09-00152-s001.zip › viruses-187399_2proofreading_supplementary/FigureS13b.jpeg]

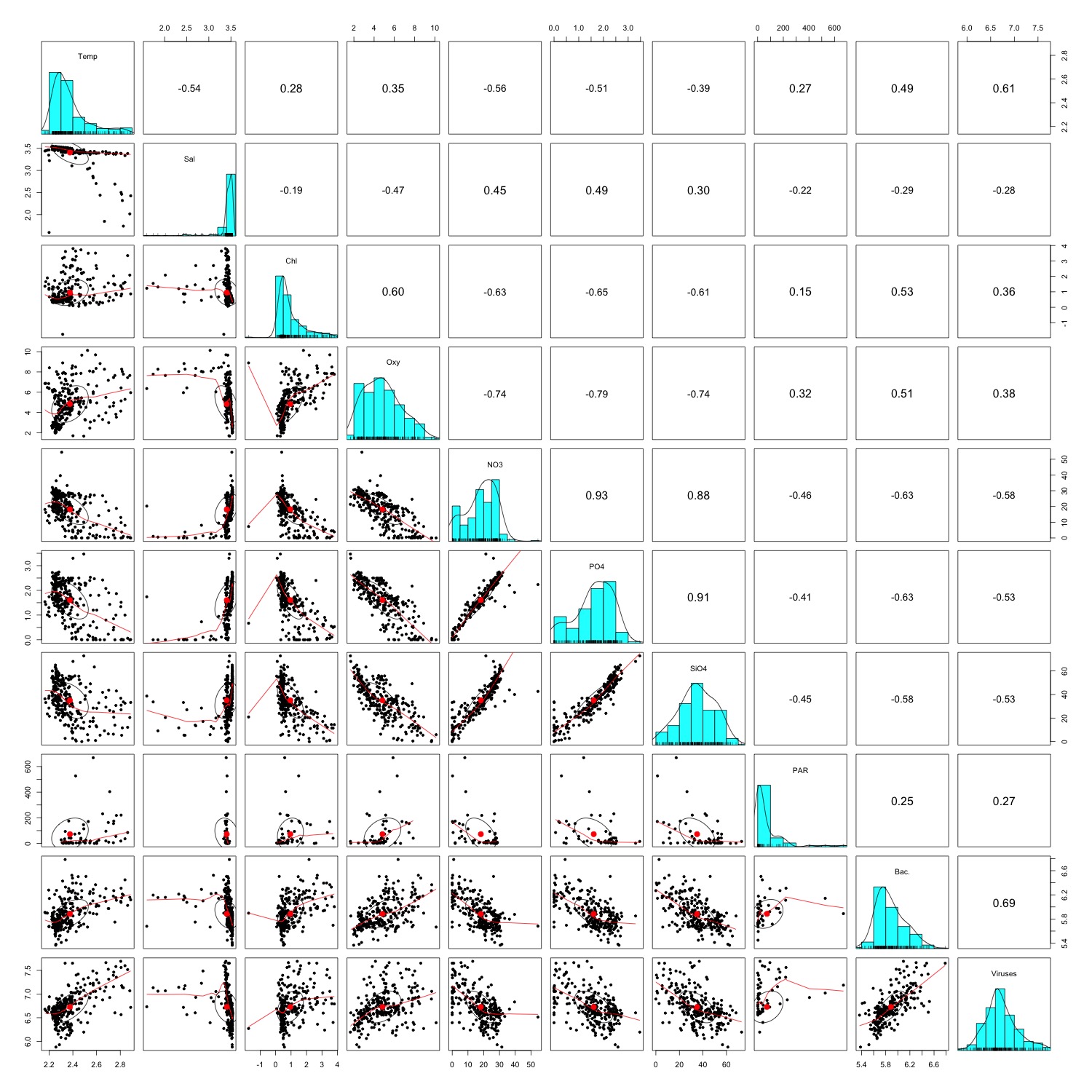

Supplement: Supplementary file 1 [file viruses-09-00152-s001.zip › viruses-187399_2proofreading_supplementary/FigureS2.jpeg]

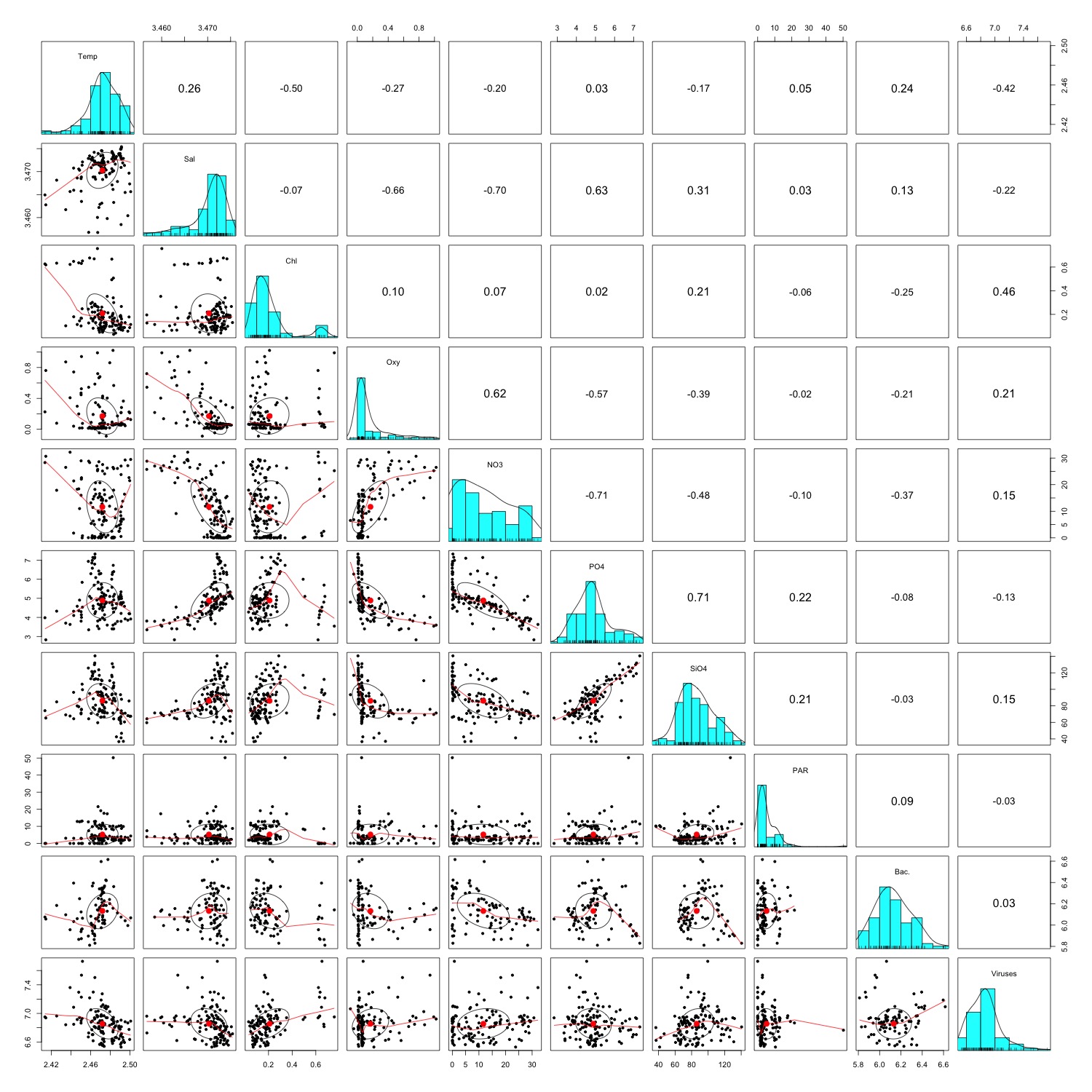

Supplement: Supplementary file 1 [file viruses-09-00152-s001.zip › viruses-187399_2proofreading_supplementary/FigureS3.jpeg]

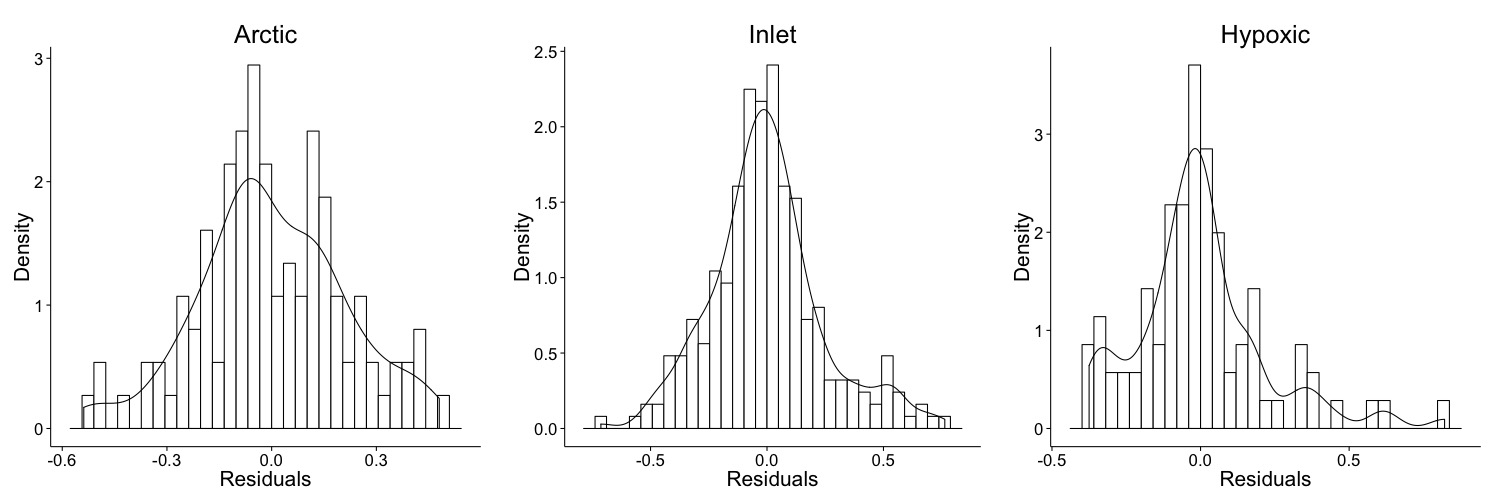

Supplement: Supplementary file 1 [file viruses-09-00152-s001.zip › viruses-187399_2proofreading_supplementary/FigureS4.jpeg]

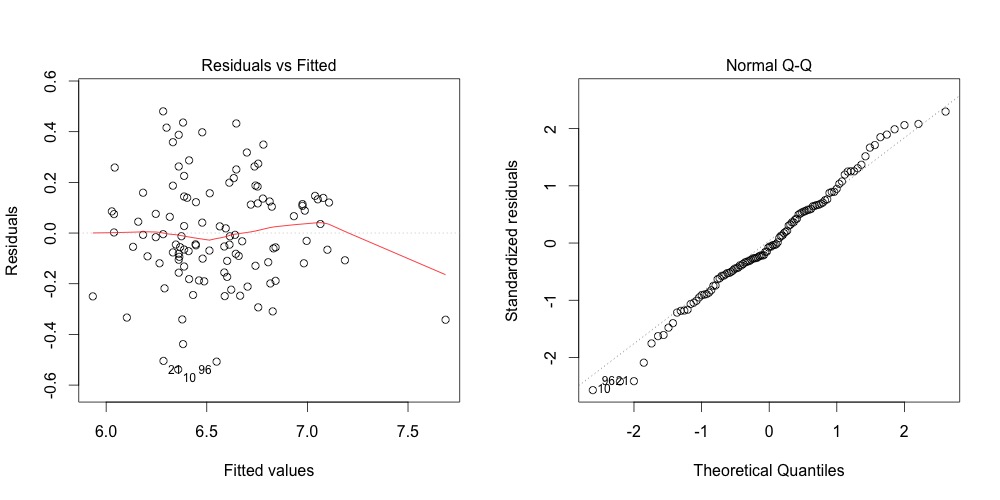

Supplement: Supplementary file 1 [file viruses-09-00152-s001.zip › viruses-187399_2proofreading_supplementary/FigureS5a.jpeg]

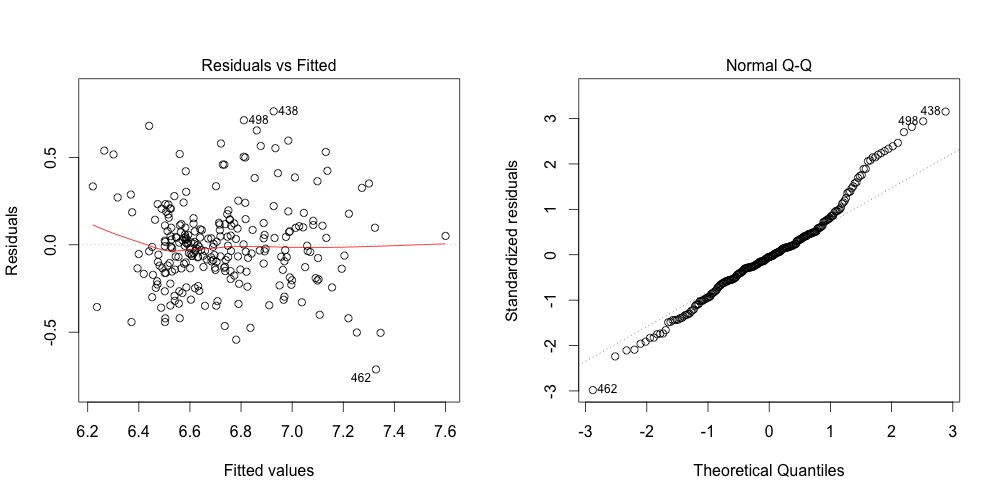

Supplement: Supplementary file 1 [file viruses-09-00152-s001.zip › viruses-187399_2proofreading_supplementary/FigureS5b.jpeg]

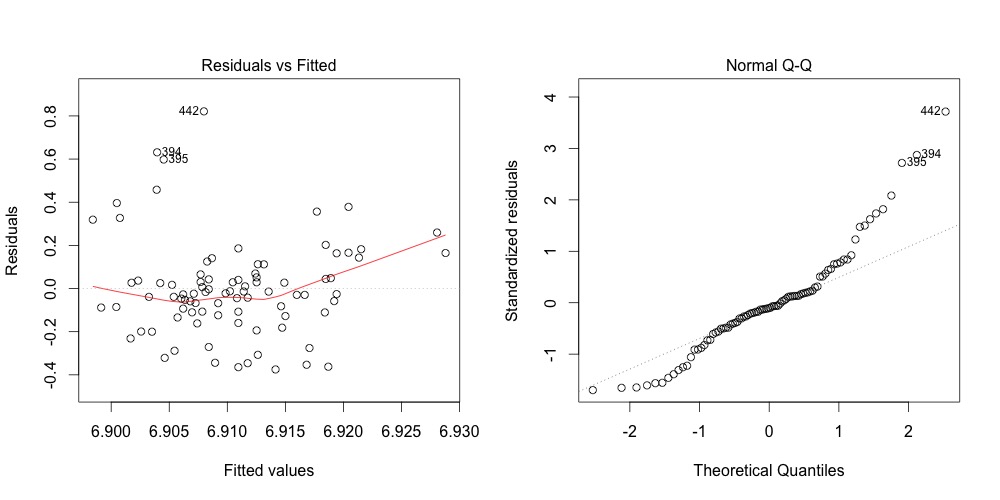

Supplement: Supplementary file 1 [file viruses-09-00152-s001.zip › viruses-187399_2proofreading_supplementary/FigureS5c.jpeg]

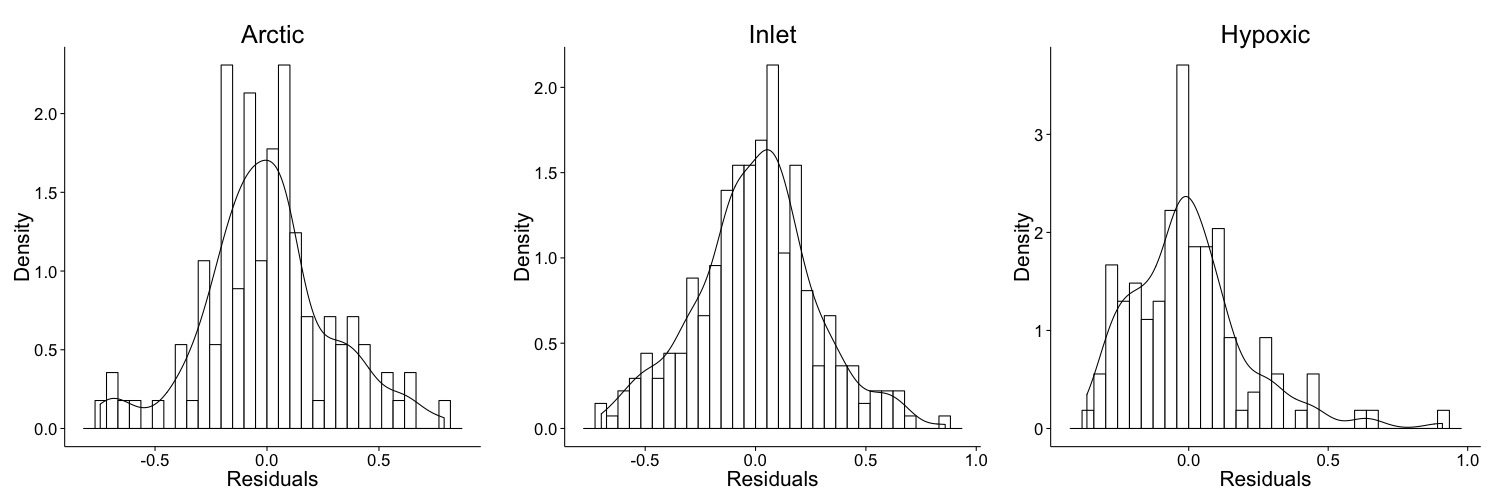

Supplement: Supplementary file 1 [file viruses-09-00152-s001.zip › viruses-187399_2proofreading_supplementary/FigureS6.jpeg]

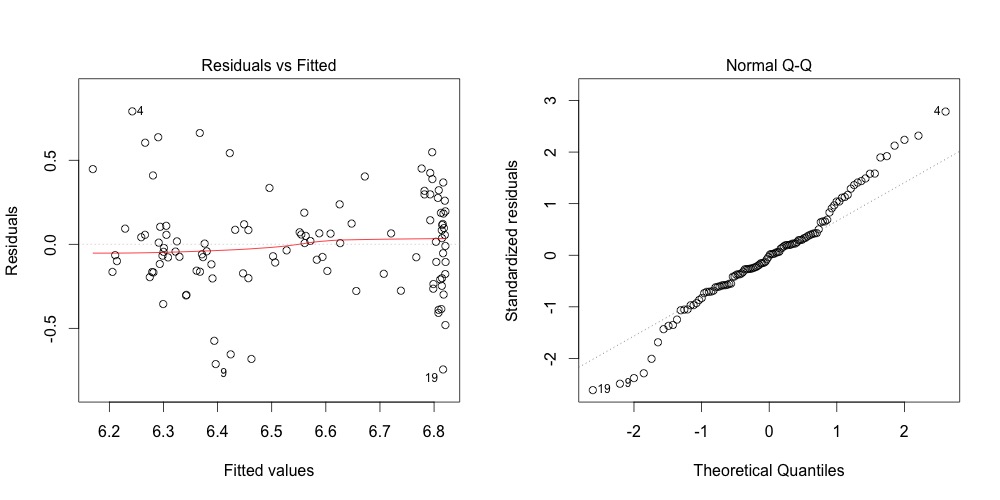

Supplement: Supplementary file 1 [file viruses-09-00152-s001.zip › viruses-187399_2proofreading_supplementary/FigureS7a.jpeg]

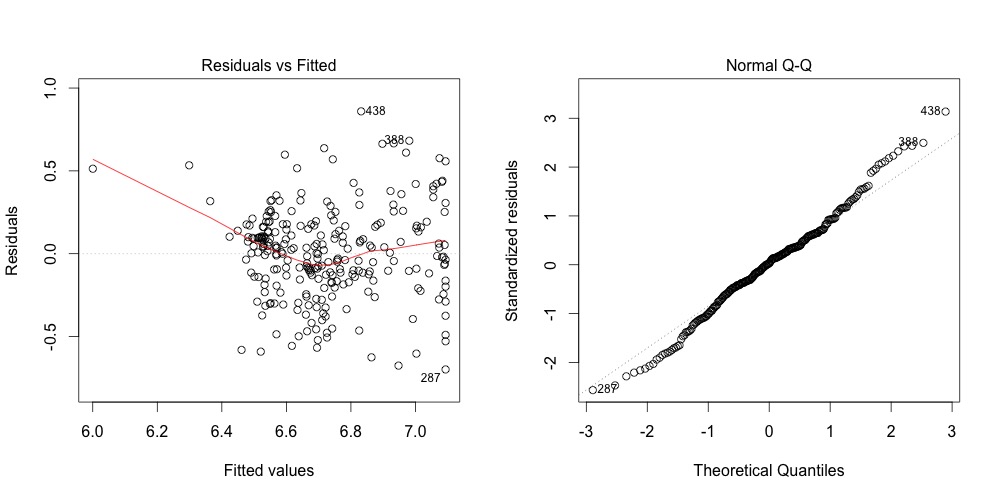

Supplement: Supplementary file 1 [file viruses-09-00152-s001.zip › viruses-187399_2proofreading_supplementary/FigureS7b.jpeg]

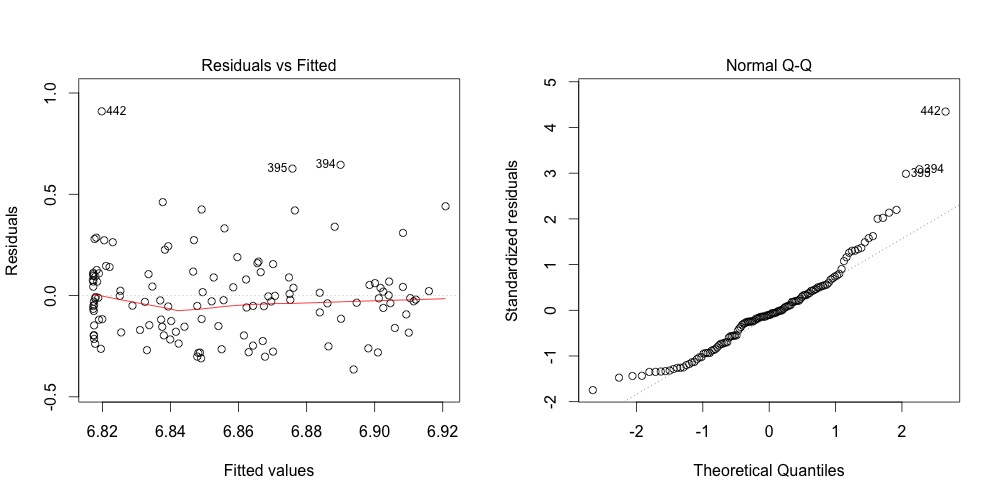

Supplement: Supplementary file 1 [file viruses-09-00152-s001.zip › viruses-187399_2proofreading_supplementary/FigureS7c.jpeg]

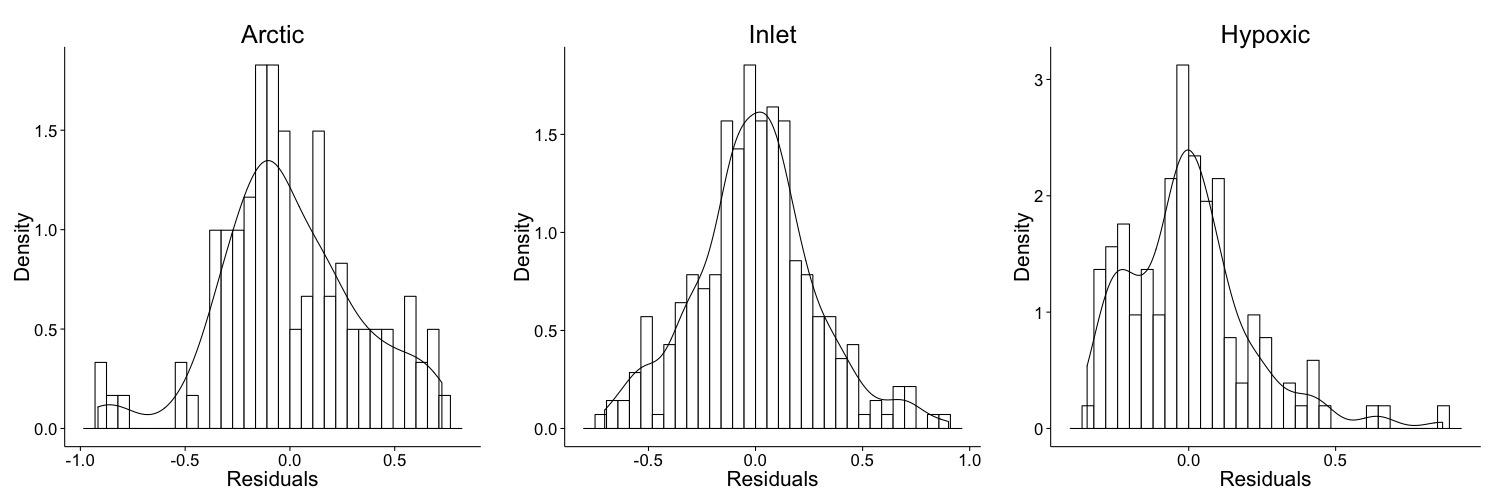

Supplement: Supplementary file 1 [file viruses-09-00152-s001.zip › viruses-187399_2proofreading_supplementary/FigureS8.jpeg]

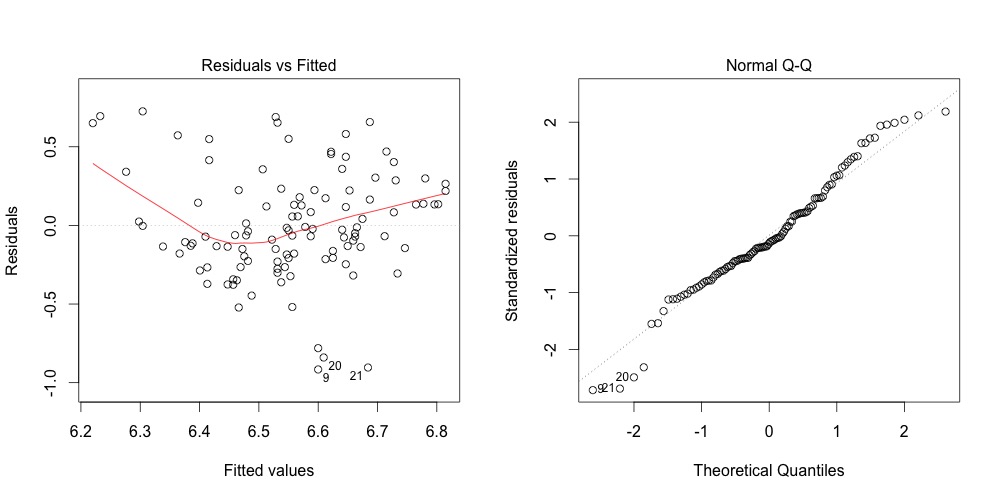

Supplement: Supplementary file 1 [file viruses-09-00152-s001.zip › viruses-187399_2proofreading_supplementary/FigureS9a.jpeg]

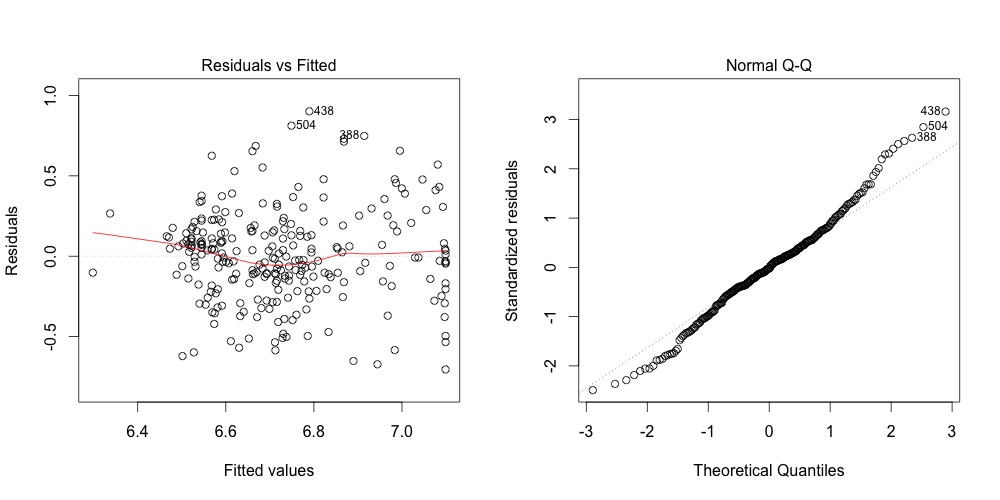

Supplement: Supplementary file 1 [file viruses-09-00152-s001.zip › viruses-187399_2proofreading_supplementary/FigureS9b.jpeg]

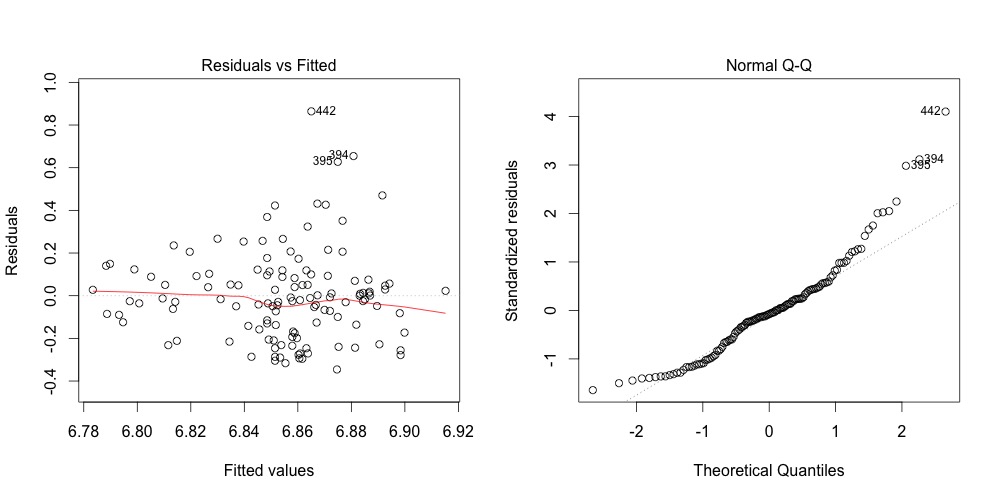

Supplement: Supplementary file 1 [file viruses-09-00152-s001.zip › viruses-187399_2proofreading_supplementary/FigureS9c.jpeg]
